# Supplementary material for: EPIC: Inferring relevant cell types for complex traits by integrating genome-wide association studies and single-cell RNA sequencing
Source: PLoS Genet. 2022 Jun 16;18(6):e1010251. doi: 10.1371/journal.pgen.1010251 (PMC9242467; doi:10.1371/journal.pgen.1010251)
Supplement: S1 Text — Supplementary materials including Note A-B, Table A-G, and Figure A-J. (DOCX) [file pgen.1010251.s001.docx]

**Supplements to "EPIC: inferring relevant cell types for complex traits by integrating genome-wide association studies and single-cell RNA sequencing"**

Rujin Wang^1^, Dan-Yu Lin^1,2,*^, Yuchao Jiang^1,2,3,*^

1. Department of Biostatistics, Gillings School of Global Public Health, University of North Carolina, Chapel Hill, NC 27599, USA.
2. Lineberger Comprehensive Cancer Center, University of North Carolina, Chapel Hill, NC 27599, USA.
3. Department of Genetics, School of Medicine, University of North Carolina, Chapel Hill, NC 27599, USA.

- To whom correspondence should be addressed: [lin@bios.unc.edu](mailto:lin@bios.unc.edu), [yuchaoj@email.unc.edu](mailto:yuchaoj@email.unc.edu).

# Supplementary Note

**Note A: Tissue-specific gene selection in bulk GTEx dataset**

We compute gene specificity score for gene $i$ and tissue $t$ as follows:

$$s_{i}^{t}=\frac{\mathrm{median}\left( e_{i}^{t} \right)-\mathrm{median}\left( e_{i}^{all} \right)}{\mathrm{IQR}\left( e_{i}^{all} \right)},$$

where $\mathrm{median}\left( e_{i}^{t} \right)$ is the median expression of gene $i$ in a particular tissue $t$; $\mathrm{median}\left( e_{i}^{all} \right)$ and $\mathrm{IQR}\left( e_{i}^{all} \right)$are the median and IQR of its expression across all samples. We define genes with a gene specificity score $s_{i}^{t}\geq5$ in any tissue $t$ as tissue-specific genes, which are selected in subsequent analyses.

**Note B: Derivation of gene-gene correlations**

We first consider a special case that each gene only contains one SNP. Let $X\sim N\left( 0,1 \right)$, $Y\sim N\left( 0,1 \right),$ and $\mathrm{cov}\left( X,Y \right)=\mathrm{cor}\left( X,Y \right)=\rho$. The goal is to compute $\mathrm{cov}(X^{2}, Y^{2})$ and $\mathrm{cor}(X^{2}, Y^{2})$.

Let $X=U_{1}$, $Y=\rho U_{1}+\sqrt{1-\rho^{2}}U_{2}$, where $U_{1},U_{2}\overset{iid}{\sim}N(0,1)$.

$$\mathrm{cov}\left( X^{2}, Y^{2} \right)=\mathrm{cov}\left( U_{1}^{2}, \left( \rho U_{1}+\sqrt{1-\rho^{2}}U_{2} \right)^{2} \right)$$

$$=\rho^{2}\mathrm{cov}\left( U_{1}^{2}, U_{1}^{2} \right)+2\rho\sqrt{1-\rho^{2}}\mathrm{cov}\left( U_{1}^{2}, U_{1}U_{2} \right)+\left( 1-\rho^{2} \right)\mathrm{cov}\left( U_{1}^{2},U_{2}^{2} \right)$$

$$=\rho^{2}\mathrm{cov}\left( U_{1}^{2}, U_{1}^{2} \right)=2\rho^{2},$$

by making use of $\mathrm{cov}\left( U_{1}, U_{1} \right)=1$, $\mathrm{cov}\left( U_{1}, U_{2} \right)=0$, and $\mathrm{cov}\left( U_{1}^{2}, U_{1}U_{2} \right)=0$.

Now we consider the generalized case. Let $X\sim\mathrm{MVN}\left( 0, I_{p} \right)$, $Y\sim\mathrm{MVN}(0, I_{q})$, and $\mathrm{cov}\left( \begin{matrix} X \\ Y \end{matrix} \right)=\mathrm{cor}\left( \begin{matrix} X \\ Y \end{matrix} \right)=R_{\left( p+q \right)\times\left( p+q \right)}=\left( \begin{matrix} I_{p} & R_{XY} \\ R_{XY}^{T} & I_{q} \end{matrix} \right)$. Suppose $U\sim\mathrm{MVN}\left( 0, I_{p+q} \right)$. We take advantage of Cholesky decomposition:

$$R=LL^{T},$$

where

$$L=\left( \begin{matrix} L_{11} & 0 & 0 & \cdots& 0 \\ L_{21} & L_{22} & 0 & \cdots& 0 \\ L_{31} & L_{32} & L_{33} & \cdots& 0 \\ \vdots& \vdots& \vdots& \vdots& \vdots\\ L_{\left( p+q \right)1} & L_{\left( p+q \right)2} & L_{\left( p+q \right)3} & \cdots& L_{\left( p+q \right)\left( p+q \right)} \end{matrix} \right).$$

Therefore, $\left( \begin{matrix} X \\ Y \end{matrix} \right)=LU$. For each $i,j,k=1,\cdots,\left( p+q \right)$ and $i\neq j\neq k$, we have $\mathrm{Var}\left( U_{i} \right)=2$, $\mathrm{cov}\left( U_{i}^{2}, U_{j}^{2} \right)=0$, $\mathrm{cov}\left( U_{i}^{2}, U_{i}U_{j} \right)=0$, $\mathrm{cov}\left( U_{i}U_{j}, U_{i}U_{j} \right)=1$, and $\mathrm{cov}\left( U_{i}U_{j}, U_{i}U_{k} \right)=0$.

$$\mathrm{cov}\left( X^{T}X, Y^{T}Y \right)=\mathrm{cov}\left( \left( L_{\left[ 1:p,\cdot\right]}U \right)^{T}\left( L_{\left[ 1:p,\cdot\right]}U \right), \left( L_{\left[ (p+1):(p+q),\cdot\right]}U \right)^{T}\left( L_{\left[ (p+1):(p+q),\cdot\right]}U \right) \right)$$

$$=\left( \sum_{i=1}^{p} L_{i1}^{2} \right)\mathrm{Var}\left( U_{1}^{2} \right)\left( \sum_{i=p+1}^{p+q} L_{i1}^{2} \right)+\cdots+\left( \sum_{i=1}^{p} L_{ip}^{2} \right)\mathrm{Var}\left( U_{p}^{2} \right)\left( \sum_{i=p+1}^{p+q} L_{ip}^{2} \right)+4\sum_{m=1}^{p-1} \sum_{n=m+1}^{p} \mathrm{cov}\left( U_{m}U_{n}, U_{m}U_{n} \right)\left( \sum_{i=1}^{p} L_{im}L_{in} \right)\left( \sum_{i=p+1}^{p+q} L_{im}L_{in} \right)$$

$$=2\left( \left( \sum_{i=1}^{p} L_{i1}^{2} \right)\left( \sum_{i=p+1}^{p+q} L_{i1}^{2} \right)+\cdots+\left( \sum_{i=1}^{p} L_{ip}^{2} \right)\left( \sum_{i=p+1}^{p+q} L_{ip}^{2} \right) \right)+4\sum_{m=1}^{p-1} \sum_{n=m+1}^{p} \left( \left( \sum_{i=1}^{p} L_{im}L_{in} \right)\left( \sum_{i=p+1}^{p+q} L_{im}L_{in} \right) \right),$$

where $L_{\left[ i:j,\cdot\right]}$ indicates the submatrix of $L$ from $i$th to $j$th row.

Specifically, given $R=\left( \begin{matrix} I_{p} & R_{XY} \\ R_{XY}^{T} & I_{q} \end{matrix} \right)$ and the Cholesky decomposition steps, $L_{\left[ 1:p,1:p \right]}=I_{p}$.

As a result,

$$\mathrm{cov}\left( X^{T}X, Y^{T}Y \right)=2\left( \left( \sum_{i=p+1}^{p+q} L_{i1}^{2} \right)+\cdots+\left( \sum_{i=p+1}^{p+q} L_{ip}^{2} \right) \right)$$

For the chi-square gene-level association statistics, let $X={R^{\left( s \right)}}^{-1/2}\hat{z}^{\left( s \right)}$, $Y={R^{\left( t \right)}}^{-1/2}\hat{z}^{\left( t \right)}$, so that $X\sim\mathrm{MVN}\left( 0, I_{K_{s}} \right)$, $Y\sim\mathrm{MVN}\left( 0, I_{K_{t}} \right)$, and

$$\mathrm{cov}\left( \begin{matrix} X \\ Y \end{matrix} \right)=\mathrm{cor}\left( \begin{matrix} X \\ Y \end{matrix} \right)=\tilde{R}_{\left( K_{s}+K_{t} \right)\times\left( K_{s}+K_{t} \right)}=\left( \begin{matrix} I_{K_{s}} & {R^{\left( s \right)}}^{-1/2}R^{\left( s,t \right)}{R^{\left( t \right)}}^{-1/2} \\ {R^{\left( t \right)}}^{-1/2}R^{\left( t,s \right)}{R^{\left( s \right)}}^{-1/2} & I_{K_{t}} \end{matrix} \right)$$

Denote $Q_{s}=\left( \hat{z}^{\left( s \right)} \right)^{T}\left( R^{\left( s \right)} \right)^{-1}\hat{z}^{\left( s \right)}$ and $Q_{t}=\left( \hat{z}^{\left( t \right)} \right)^{T}\left( R^{\left( t \right)} \right)^{-1}\hat{z}^{\left( t \right)}$. We perform Cholesky decomposition on $\tilde{R}=LL^{T}$ and obtain

$$\mathrm{cov}\left( Q_{s}, Q_{t} \right)=2\left( \sum_{j=1}^{K_{s}} \sum_{i=1}^{K_{s}+K_{t}} L_{ij}^{2} \right)$$

As a result,

$$\rho_{st}=cor\left( Q_{s}, Q_{t} \right)=\frac{\sum_{j=1}^{K_{s}} \sum_{i=1}^{K_{s}+K_{t}} L_{ij}^{2}}{\sqrt{K_{s}K_{t}}},$$

where $L_{ij}$’s are entries of a lower triangular matrix $L$ such that $\tilde{R}_{\left( K_{s}+K_{t} \right)\times\left( K_{s}+K_{t} \right)}=LL^{T}$.

# Supplementary Tables

**Table A: Summary of GWAS studies and transcriptomic studies.**

**(A)** Summary information for nine GWAS studies

| Phenotype | # of subjects | Reference | URL |
| --- | --- | --- | --- |
| Low-density lipoprotein cholesterol (LDL) | 188,577 | Willer et al., 2013 [1] | <http://csg.sph.umich.edu/willer/public/lipids2013/> |
| High-density lipoprotein cholesterol (HDL) | 188,577 | Willer et al., 2013 [1] | <http://csg.sph.umich.edu/willer/public/lipids2013/> |
| Total cholesterol (TC) | 188,577 | Willer et al., 2013 [1] | <http://csg.sph.umich.edu/willer/public/lipids2013/> |
| Triglyceride levels (TG) | 188,577 | Willer et al., 2013 [1] | <http://csg.sph.umich.edu/willer/public/lipids2013/> |
| Schizophrenia (SCZ) | 79,845 | Ripke et al., 2014 [2] | <https://www.med.unc.edu/pgc/download-results/> |
| Schizophrenia (SCZ2) | 105,318 | Pardinas et al., 2018 [3] | <https://walters.psycm.cf.ac.uk/> |
| Bipolar disorder (BIP) | 51,710 | Stahl et al., 2019 [4] | <https://www.med.unc.edu/pgc/download-results/> |
| Schizophrenia and bipolar disorder (SCZBIP) | 107,620 | Ruderfer et al., 2018 [5] | <https://www.med.unc.edu/pgc/download-results/> |
| Type 2 diabetes (T2Db) | 898,130 | Mahajan et al., 2018b [6] | <http://diagram-consortium.org/downloads.html> |

**(B)** Summary information for transcriptomic studies

| Name | Tissue or cell type | Technology | Reference |
| --- | --- | --- | --- |
| GTEx bulk | 45 tissues from 980 donors | RNA-seq | GTEx Consortium [7] |
| Pancreatic islet scRNA-seq: Baron | 13 cell types from 3 healthy donors | InDrop | Baron et al. [8] |
| Pancreatic islet scRNA-seq: Segerstolpe | 12 cell types from 6 healthy donors | SMART-Seq2 | Segerstolpe et al. [9] |
| GTEx scRNA-seq | 10 brain cell types from 5 donors | DroNc-seq | Habib et al. [10] |

**Table B. Gene-level association testing results of eight GWAS traits.** A final set of 8,708 genes are retained. We selected a list of risk genes within implicated genome-wide significant loci that were reported in the original GWAS [1, 2, 4-6] for each trait. Significant gene-level associations were detected between all lipid traits and variants in *APOB*, *APOE*, and *CETP*. Meanwhile, *PCSK9*, *ABCG5,* and *ABCG8* exhibited significant associations with LDL and TC. For neuropsychiatric disorders, we examined genes that are relevant to the etiology of schizophrenia, including genes that are targets of therapeutic drugs (*DRD2* and *GRM3*), genes that participate in neuronal calcium signaling (*CACNA1I*), and genes that are involved in synaptic function (*CNTN4* and *SNAP91*) and other neuronal pathways (*FXR1*, *CHRNA3*, *CHRNB4*, and *HCN1*). The numbers of common and rare SNPs within each gene are shown. EPIC's chi-square test approach demonstrates higher power than MAGMA and ACAT. Zero $p$-values are set to $1.00\times{10}^{-300}$ for all methods.

(A) LDL: gene-level association testing


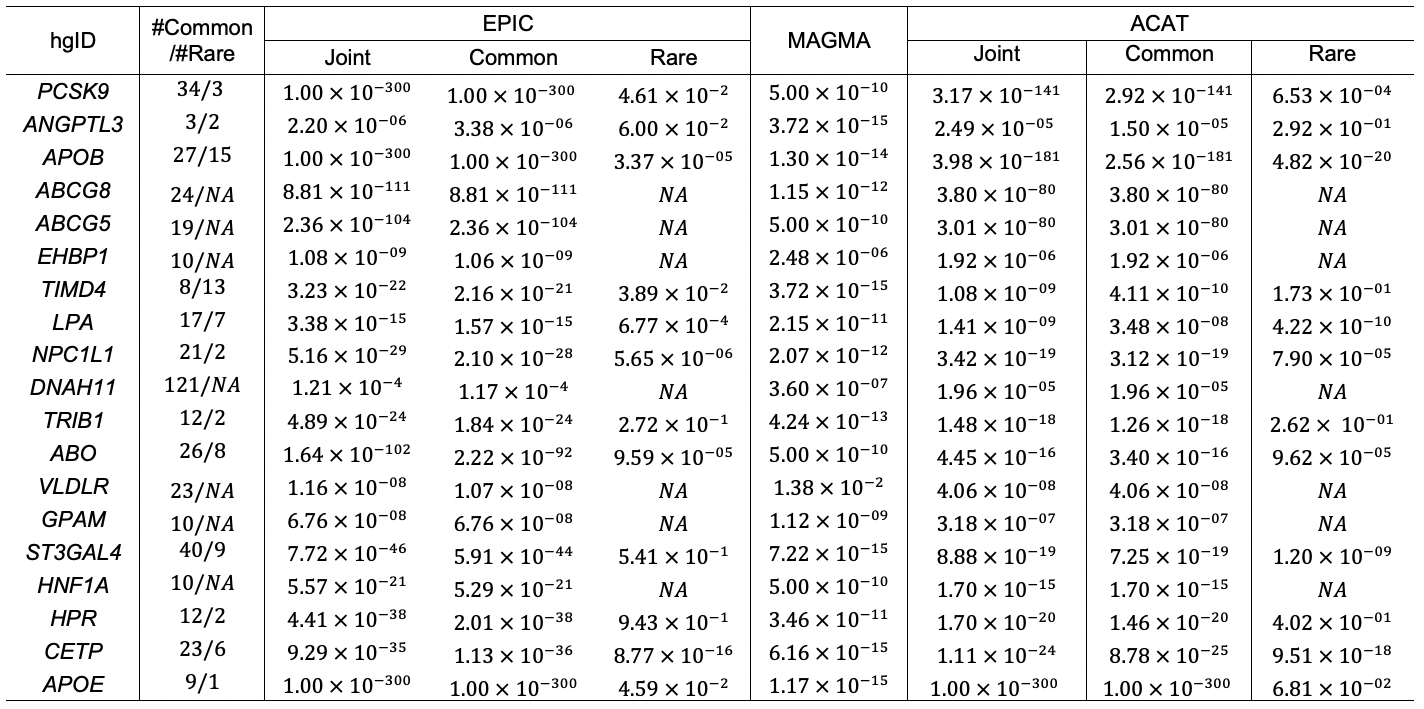


(B) HDL: gene-level association testing


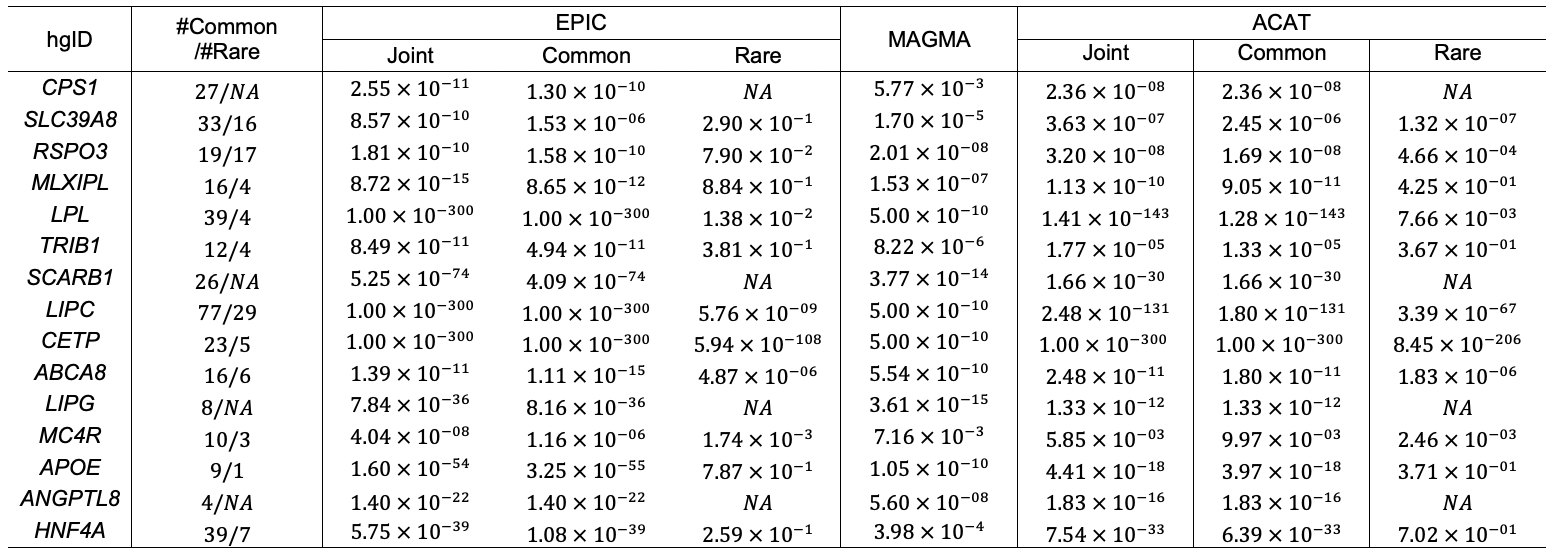


(C) TC: gene-level association testing


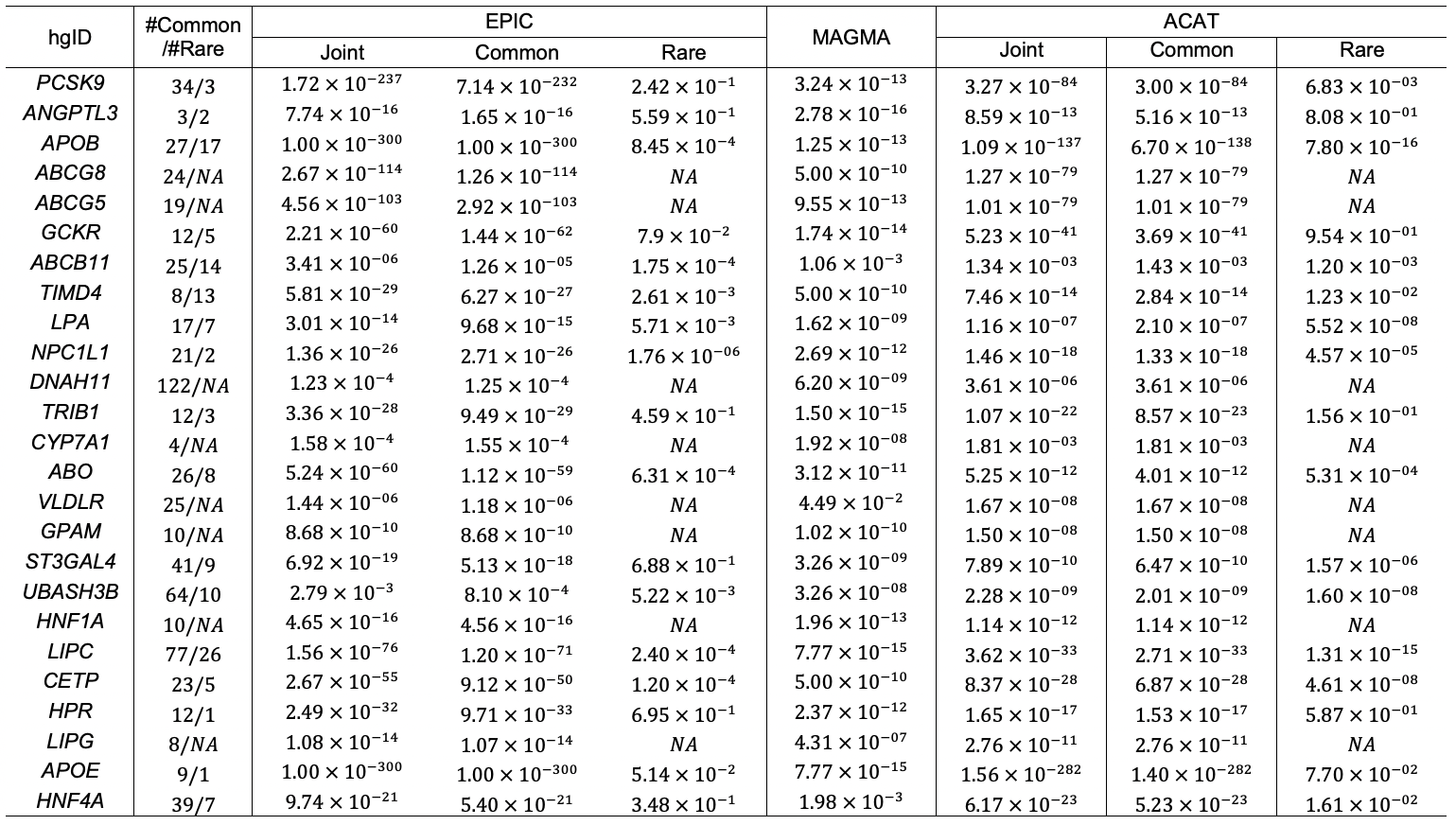


(D) TG: gene-level association testing


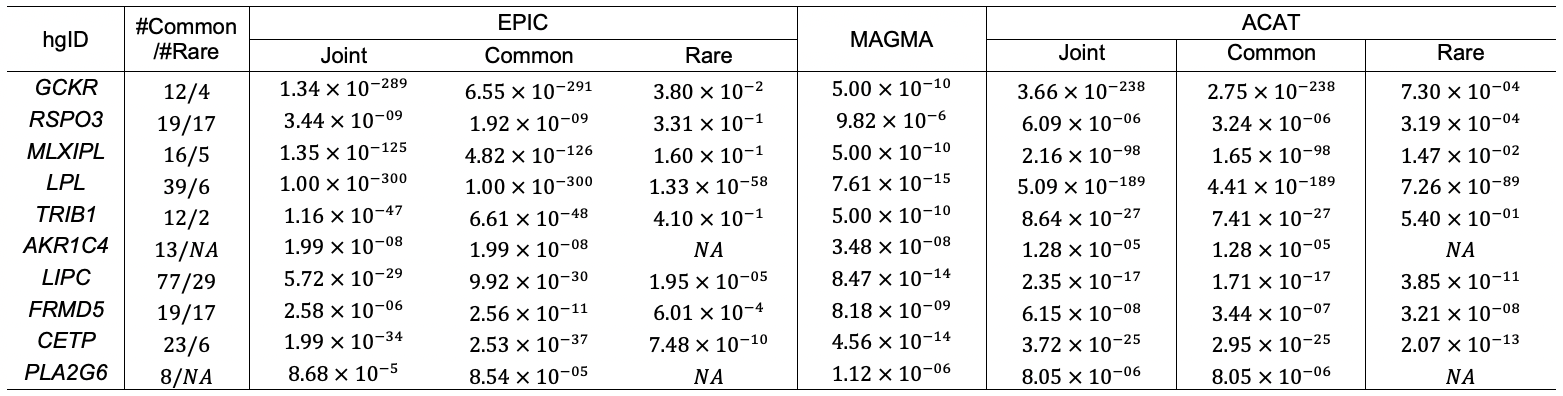


(E) SCZ: gene-level association testing


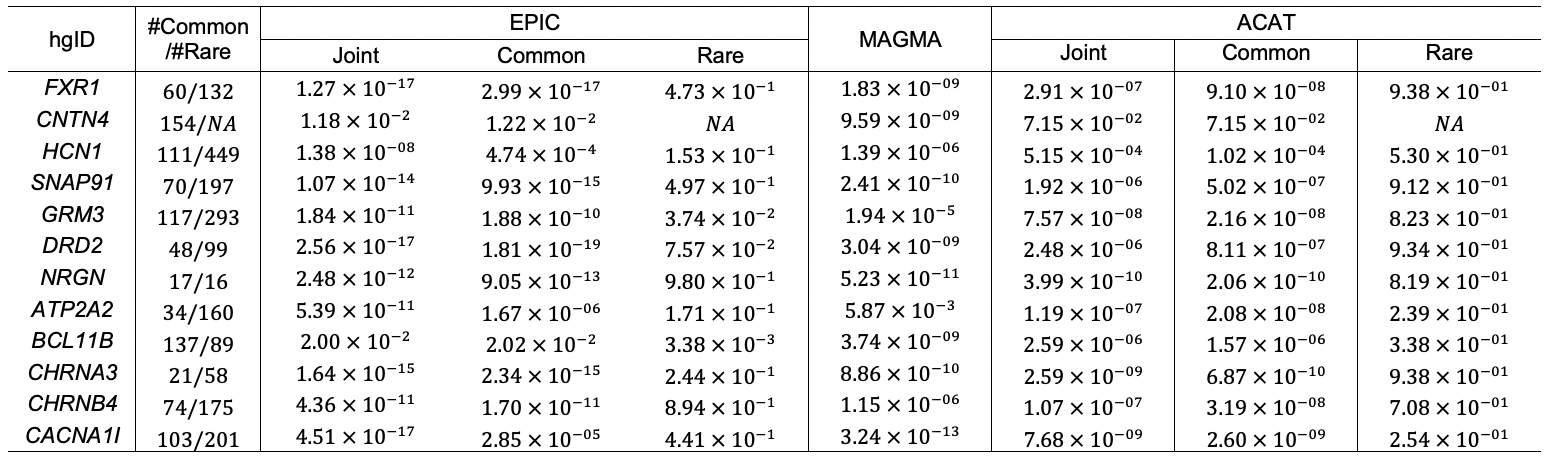


(F) BIP: gene-level association testing


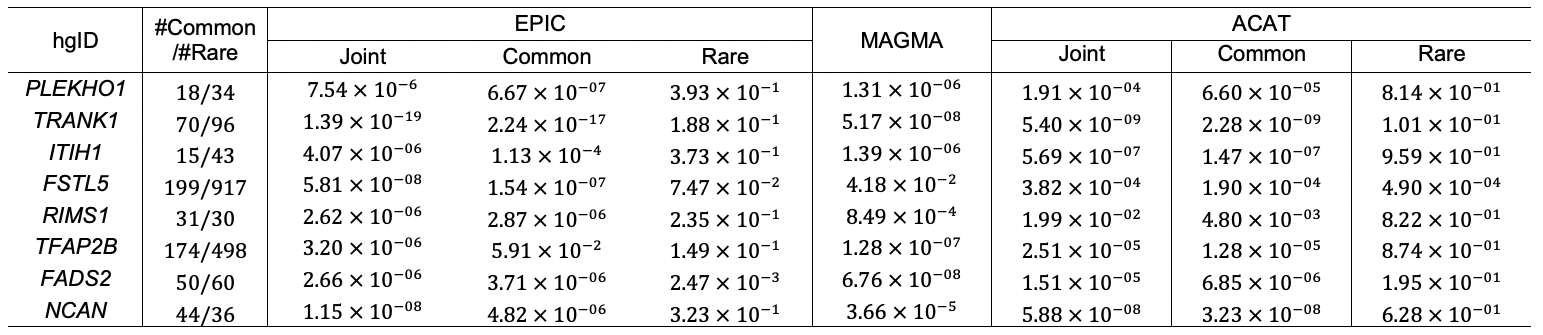


(G) SCZBIP: gene-level association testing


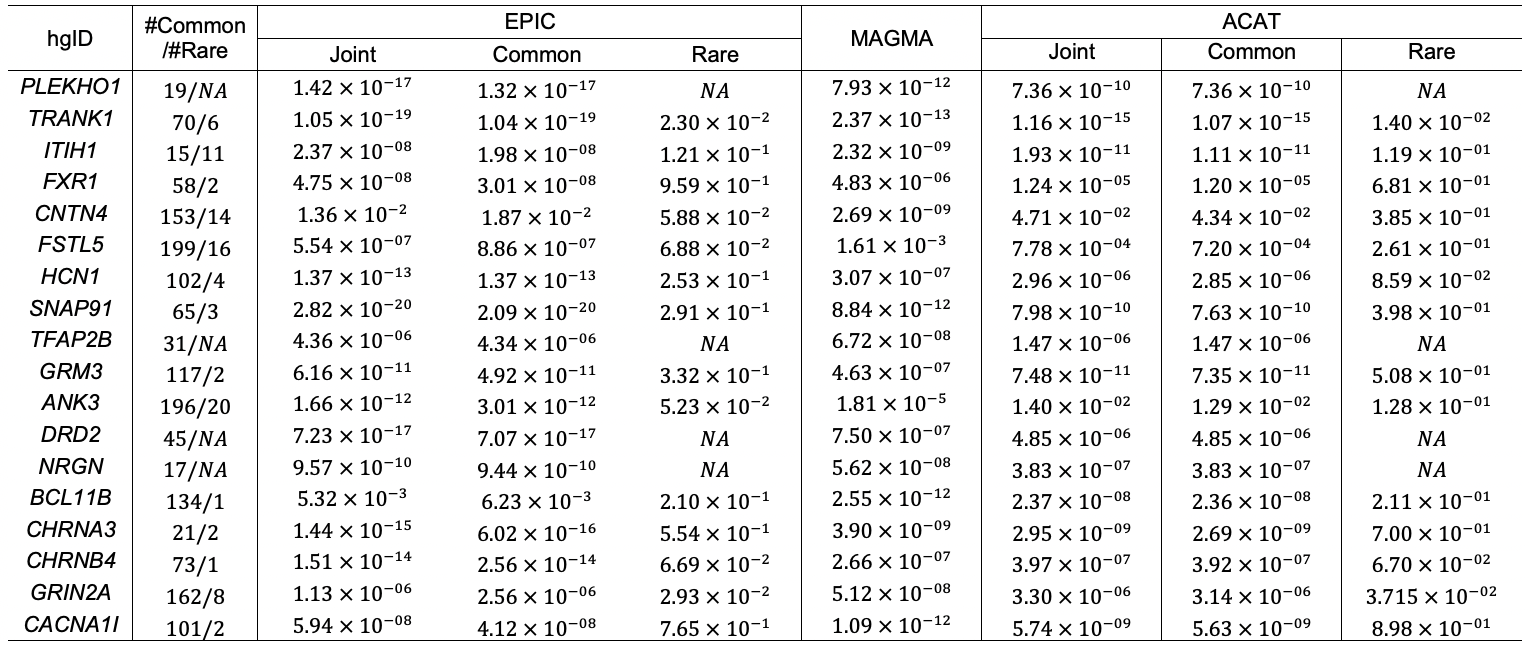


(H) T2Db: gene-level association testing


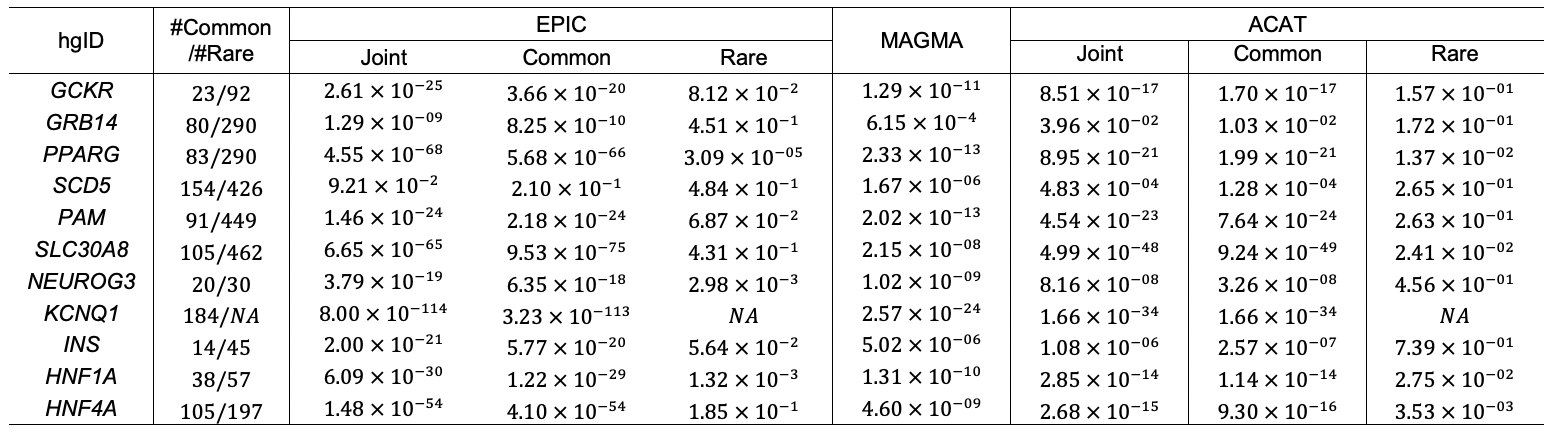


**Table C. DAVID enrichment analysis using significantly associated genes detected EPIC but not by MAGMA.** Enriched GO biological processes with p-value < 0.05 are reported, and they are relevant to the traits of interest.

(A)

| Trait | Term | Count | p-value |
| --- | --- | --- | --- |
| LDL | quaternary ammonium group transport | 2 | $8.3\times{10}^{-3}$ |
|  | negative regulation of fibrinolysis | 2 | $1.6\times{10}^{-2}$ |
|  | cellular glucose homeostasis | 2 | $2.2\times{10}^{-2}$ |
|  | organic cation transport | 2 | $2.4\times{10}^{-2}$ |
|  | positive regulation of dendrite development | 2 | $2.5\times{10}^{-2}$ |
|  | metabolic process | 3 | $3.0\times{10}^{-2}$ |
|  | signal transduction | 6 | $3.5\times{10}^{-2}$ |

(B)

| Trait | Term | Count | p-value |
| --- | --- | --- | --- |
| HDL | adenylate cyclase-modulating G-protein coupled receptor signaling pathway | 3 | $2.2\times{10}^{-3}$ |
|  | diet induced thermogenesis | 2 | $1.7\times{10}^{-2}$ |
|  | negative regulation of cell growth | 3 | $2.2\times{10}^{-2}$ |
|  | lipid metabolic process | 3 | $3.6\times{10}^{-2}$ |

(C)

| Trait | Term | Count | p-value |
| --- | --- | --- | --- |
| TC | negative regulation of fibrinolysis | 2 | $1.3\times{10}^{-3}$ |
|  | positive regulation of dendrite development | 2 | $2.1\times{10}^{-2}$ |
|  | multicellular organism development | 4 | $3.0\times{10}^{-2}$ |
|  | triglyceride homeostasis | 2 | $3.4\times{10}^{-2}$ |

(D)

| Trait | Term | Count | p-value |
| --- | --- | --- | --- |
| TG | chemical synaptic transmission | 4 | $5.9\times{10}^{-3}$ |
|  | ion transport | 3 | $1.6\times{10}^{-2}$ |
|  | bile acid and bile salt transport | 2 | $4.1\times{10}^{-2}$ |
|  | proteolysis | 4 | $4.1\times{10}^{-2}$ |
|  | regulation of apoptotic process | 3 | $4.3\times{10}^{-2}$ |

(E)

| Trait | Term | Count | p-value |
| --- | --- | --- | --- |
| SCZ | central nervous system development | 6 | $5.7\times{10}^{-4}$ |
|  | neuron fate specification | 3 | $2.7\times{10}^{-3}$ |
|  | neuron migration | 5 | $2.9\times{10}^{-3}$ |
|  | subpallium neuron fate commitment | 2 | $1.1\times{10}^{-2}$ |
|  | potassium ion export across plasma membrane | 2 | $2.2\times{10}^{-2}$ |
|  | nervous system development | 6 | $2.3\times{10}^{-2}$ |
|  | calcium ion transmembrane transport | 4 | $2.9\times{10}^{-2}$ |
|  | sensory perception of bitter taste | 2 | $3.3\times{10}^{-2}$ |
|  | sensory perception of sweet taste | 2 | $3.3\times{10}^{-2}$ |
|  | sperm motility | 3 | $3.7\times{10}^{-2}$ |
|  | transition between fast and slow fiber | 2 | $3.9\times{10}^{-2}$ |
|  | neuroepithelial cell differentiation | 2 | $3.9\times{10}^{-2}$ |
|  | clustering of voltage-gated sodium channels | 2 | $3.9\times{10}^{-2}$ |
|  | sensory perception of sound | 4 | $3.9\times{10}^{-2}$ |
|  | response to mechanical stimulus | 3 | $4.3\times{10}^{-2}$ |
|  | endoplasmic reticulum tubular network organization | 2 | $4.4\times{10}^{-2}$ |
|  | synapse assembly | 3 | $4.6\times{10}^{-2}$ |
|  | potassium ion export | 2 | $4.9\times{10}^{-2}$ |
|  | membrane repolarization | 2 | $4.9\times{10}^{-2}$ |

(F)

| Trait | Term | Count | p-value |
| --- | --- | --- | --- |
| SCZBIP | feeding behavior | 3 | $1.1\times{10}^{-2}$ |
|  | central nervous system development | 4 | $1.3\times{10}^{-2}$ |
|  | sensory perception of sound | 4 | $1.7\times{10}^{-2}$ |
|  | positive regulation of gene expression | 5 | $2.3\times{10}^{-2}$ |
|  | positive regulation of GTPase activity | 7 | $2.8\times{10}^{-2}$ |
|  | hepatocyte differentiation | 2 | $4.0\times{10}^{-2}$ |
|  | brain development | 4 | $4.3\times{10}^{-2}$ |
|  | response to pH | 2 | $4.8\times{10}^{-2}$ |

(G)

| Trait | Term | Count | p-value |
| --- | --- | --- | --- |
| T2Db | ion transport | 7 | $1.2\times{10}^{-3}$ |
|  | anatomical structure morphogenesis | 6 | $1.7\times{10}^{-3}$ |
|  | signal transduction | 22 | $2.5\times{10}^{-3}$ |
|  | negative regulation of insulin secretion | 4 | $2.9\times{10}^{-3}$ |
|  | protein heterooligomerization | 5 | $3.5\times{10}^{-3}$ |
|  | establishment or maintenance of transmembrane electrochemical gradient | 3 | $6.3\times{10}^{-3}$ |
|  | microtubule-based movement | 5 | $6.9\times{10}^{-3}$ |
|  | neuron fate specification | 3 | $7.3\times{10}^{-3}$ |
|  | oxygen transport | 3 | $8.3\times{10}^{-3}$ |
|  | transport | 9 | $1.6\times{10}^{-2}$ |
|  | positive regulation of transcription, DNA-templated | 11 | $2.2\times{10}^{-2}$ |
|  | neurotransmitter transport | 3 | $2.4\times{10}^{-2}$ |
|  | potassium ion transmembrane transport | 5 | $2.6\times{10}^{-2}$ |
|  | ATP hydrolysis coupled transmembrane transport | 2 | $3.7\times{10}^{-2}$ |
|  | metaphase/anaphase transition of mitotic cell cycle | 2 | $3.7\times{10}^{-2}$ |
|  | potassium ion transport | 4 | $4.1\times{10}^{-2}$ |
|  | response to zinc ion | 3 | $4.4\times{10}^{-2}$ |
|  | ion transmembrane transport | 6 | $4.6\times{10}^{-2}$ |

**Table D. Top three tissue types identified using rare variants only in the GTEx bulk RNA-seq data.** We considered rare variants only and recovered the gene-level chi-square association test statistic from the burden test. Gene-gene correlations were retrieved from the joint analysis of common and rare variants with sliding windows. We identified three top tissue types using the framework of tissue-specific enrichment analysis. The asterisk (*) indicates statistical significance at the significance level 0.05 with Bonferroni correction.

| Trait | Top 1 relevant tissue  for rare variants | Top 2 relevant tissue  for rare variants | Top 3 relevant tissue  for rare variants |
| --- | --- | --- | --- |
| LDL | Liver* | Small intestine | Colon transverse |
| HDL | Liver | Adrenal gland | Spleen |
| TC | Liver* | Small intestine | Colon transverse |
| TG | Liver | Thyroid | Pituitary |
| SCZ | Brain cerebellum | Brain cerebellar hemisphere | Heart left ventricle |
| BIP | Brain frontal cortex* | Brain cortex | Brain anterior |
| SCZBIP | Brain frontal cortex* | Brain cortex* | Brain anterior* |
| T2Db | Brain frontal cortex* | Brain cortex* | Brain cerebellar hemisphere* |

**Table E: Power evaluation for gene-level association testing by EPIC and MAGMA.** To assess EPIC’s power for gene-level association testing, we generated dichotomous phenotypes as a function of the SNP-level genotypes with varied proportions of causal variants, effect sizes, and directions of effects. We then computed the summary statistics for each SNP, which were used as input for EPIC. The proportion of causal variants was set to be 5%, 20%, and 50%, representative of both of sparse and dense signals; effect sizes, as well as directions of effects, were also varied. Altogether, we had a total of 30 simulation configurations for each of the 100 genes. Empirical power was estimated as the proportion of $p$-values less than $\alpha={10}^{-6}$. The simulation was repeated 1,000 times to allow for standard error estimates. Our results suggest that under 30 different simulation configurations EPIC is more powerful than MAGMA and that the power gain is substantial when the signals are sparse or in different directions.

**Table F: Evaluation of computational efficiency.** For the GTEx RNA-seq analysis with 45 tissues and 8,708 genes, computational time is reported for three traits (LDL, SCZ, T2Db) from different GWASs with different numbers of SNPs. Time unit is hour (h). Jobs were run with 80GB RAM on a high-performance cluster. For EPIC, we reported processing time on estimating POET shrinkage and calculating gene-gene correlations. For MAGMA, we reported time on gene-level association testing. For RolyPoly, we reported time of: (i) linking SNPs to genes, and (ii) the bootstrapping inference. For LDSC-SEG, we reported processing time of: (i) making gene annotations and computing LD scores for each tissue-chromosome pair, and (ii) the final tissue-specific analysis. We evaluate the computing time chromosome by chromosome (indicated with asterisks) if parallel computing is allowed; the running time is calculated by taking the mean.

|  | LDL | SCZ | T2Db |
| --- | --- | --- | --- |
| EPIC (*) | 0.23 | 2.20 | 2.20 |
| MAGMA | 0.11 | 0.16 | 0.38 |
| RolyPoly:link (*) | 0.42 | 6.70 | 6.00 |
| RolyPoly:inference | 0.13 | 0.72 | 0.47 |
| LDSC-SEG:anno+ldscore (*) | 1.98 | | |
| LDSC-SEG:cts | 0.70 | 0.70 | 0.90 |

**Table G: Number of common and rare variants from GWAS summary statistics with different thresholds.** We report the number of common and rare SNPs / the number of genes (average number of SNPs per gene) with different MAF and MAC thresholds. For rare variants, the upper bound of inclusion is controlled by MAF while the lower bound is determined by MAC. Rare variants with MAC less than 20 are removed from the analysis by default.

**(A)** Number of rare variants with different thresholds of MAF (upper bound)

| Trait | MAF <= 1% | MAF <= 0.1% |
| --- | --- | --- |
| LDL | 97,915 / 20,187 (5.5) | 90,528 / 19,550 (5.2) |
| HDL | 99,189 / 20,283 (5.6) | 90,918 / 19,575 (5.3) |
| TC | 98,930 / 20,315 (5.5) | 90,759 / 19,617 (5.2) |
| TG | 98,125 / 20,128 (5.5) | 90,548 / 19,476 (5.3) |
| SCZ | 1,959,467 / 30,935 (73.0) | 550,279 / 29,784 (21.3) |
| BIP | 1,487,888 / 30,878 (55.6) | 504 / 550 (1.1) |
| SCZBIP | 27,748 / 10,910 (2.9) | 0 / 0 (0) |
| T2Db | 3,224,387 / 30,991 (120.3) | 1,868,536 / 30,919 (69.6) |

**(B)** Number of rare variants with different thresholds of MAF (upper bound) and MAC filtering (lower bound)

| Trait | MAF <= 1% | MAF <= 0.1% |
| --- | --- | --- |
| LDL | 44,412 / 3,164 (14.8) | 0 / 0 (0) |
| HDL | 47,651 / 3,437 (14.7) | 0 / 0 (0) |
| TC | 47,033 / 3,406 (14.6) | 0 / 0 (0) |
| TG | 44,812 / 3,204 (14.8) | 0 / 0 (0) |
| SCZ | 1,959,449 / 30,905 (73.1) | 548,393 / 28,364 (22.2) |
| BIP | 1,487,888 / 30,878 (55.6) | 503 / 549 (1.1) |
| SCZBIP | 27,748 / 10,910 (2.9) | 0 / 0 (0) |
| T2Db | 3,224,317 / 30,918 (120.5) | 1,867,997 / 30,613 (70.2) |

**(C)** Number of pruned-in common variants with different thresholds of MAF (lower bound)

| Trait | MAF > 1% | MAF > 0.1% |
| --- | --- | --- |
| LDL | 221,998 / 27,288 (9.2) | 225,222 / 27,337 (9.3) |
| HDL | 223,456 / 27,320 (9.2) | 227,066 / 27,378 (9.3) |
| TC | 223,429 / 27,322 (9.2) | 226,991 / 27,379 (9.3) |
| TG | 222,154 / 27,290 (9.2) | 225,444 / 27,341 (9.3) |
| SCZ | 1,114,970 / 31,030 (41.1) | 1,991,247 / 31,086 (73.5) |
| BIP | 1,086,963 / 31,030 (40.1) | 1,962,422 / 31,096 (72.5) |
| SCZBIP | 1,087,425 / 30,980 (40.2) | 1,223,597 / 31,003 (45.2) |
| T2Db | 1,068,216 / 30,792 (39.7) | 2,052,221 / 30,935 (76.3) |

# Supplementary Figures

**Fig A. Gene-level quantile-quantile plots for eight GWAS traits.** EPIC achieves higher power than MAGMA and ACAT in the gene-level association test. Due to the negative log transformation, the $p$-values along the axes are not uniformly distributed but are concentrated near the origin. The observed $p$-values ranging from 0.05 to 1 agree with the corresponding expected $p$-values.

**
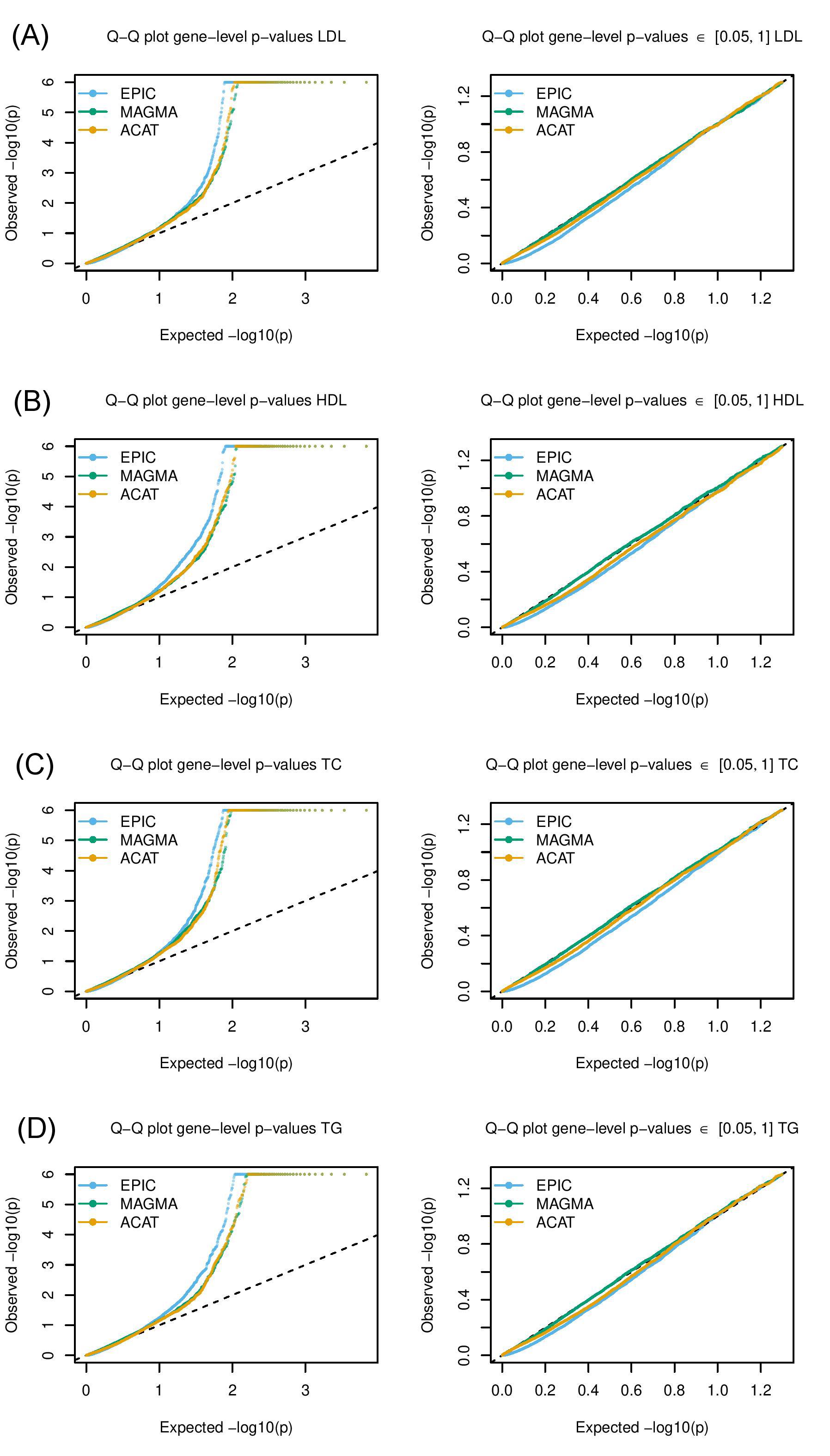
**

**
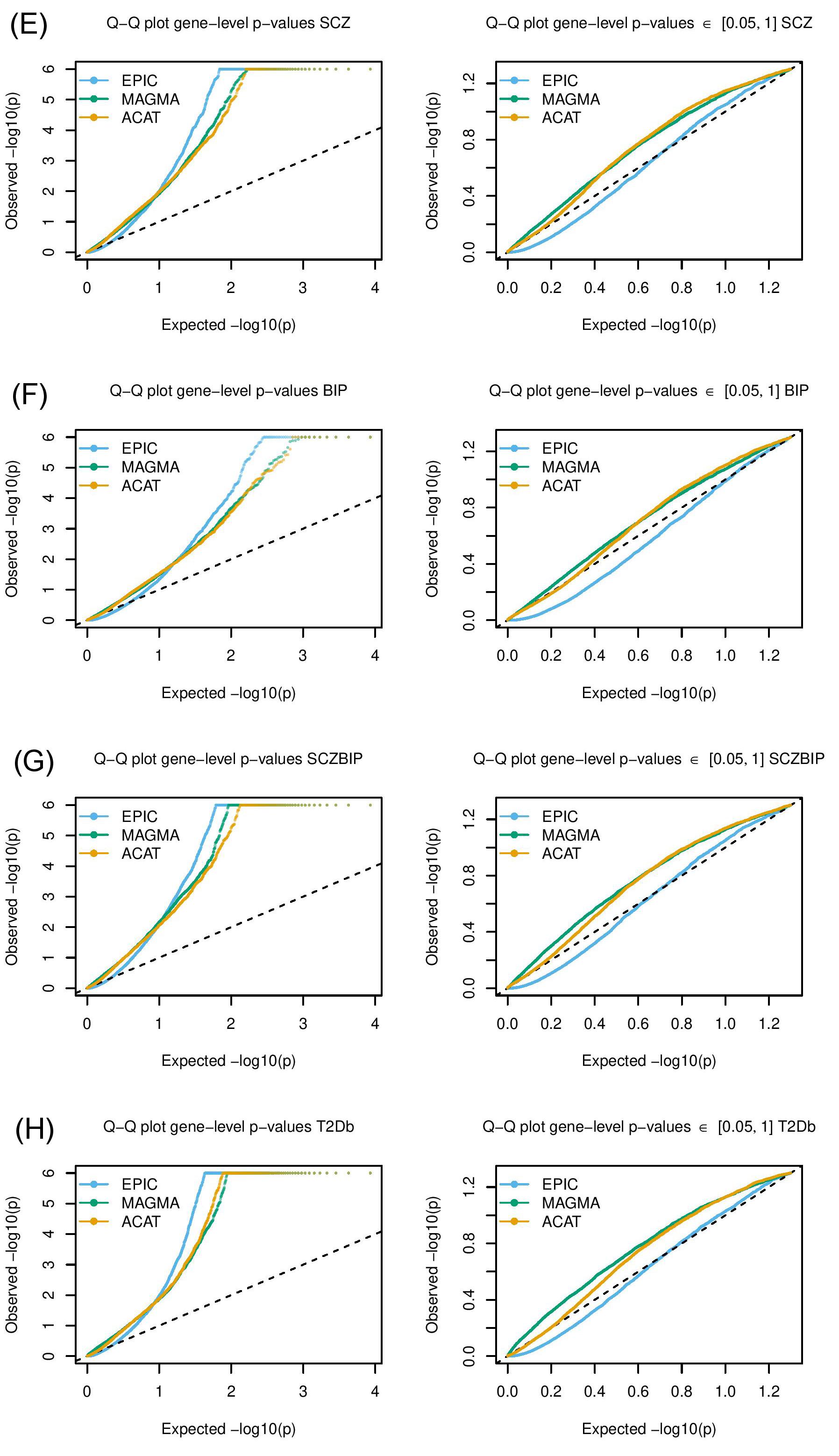
**

**Fig B. Venn diagram of significant genes associated with eight GWAS traits by EPIC and MAGMA.** EPIC detected more significantly associated genes compared to MAGMA. The additional genes detected by EPIC are enriched for terms relevant to the trait of interests via DAVID analyses.


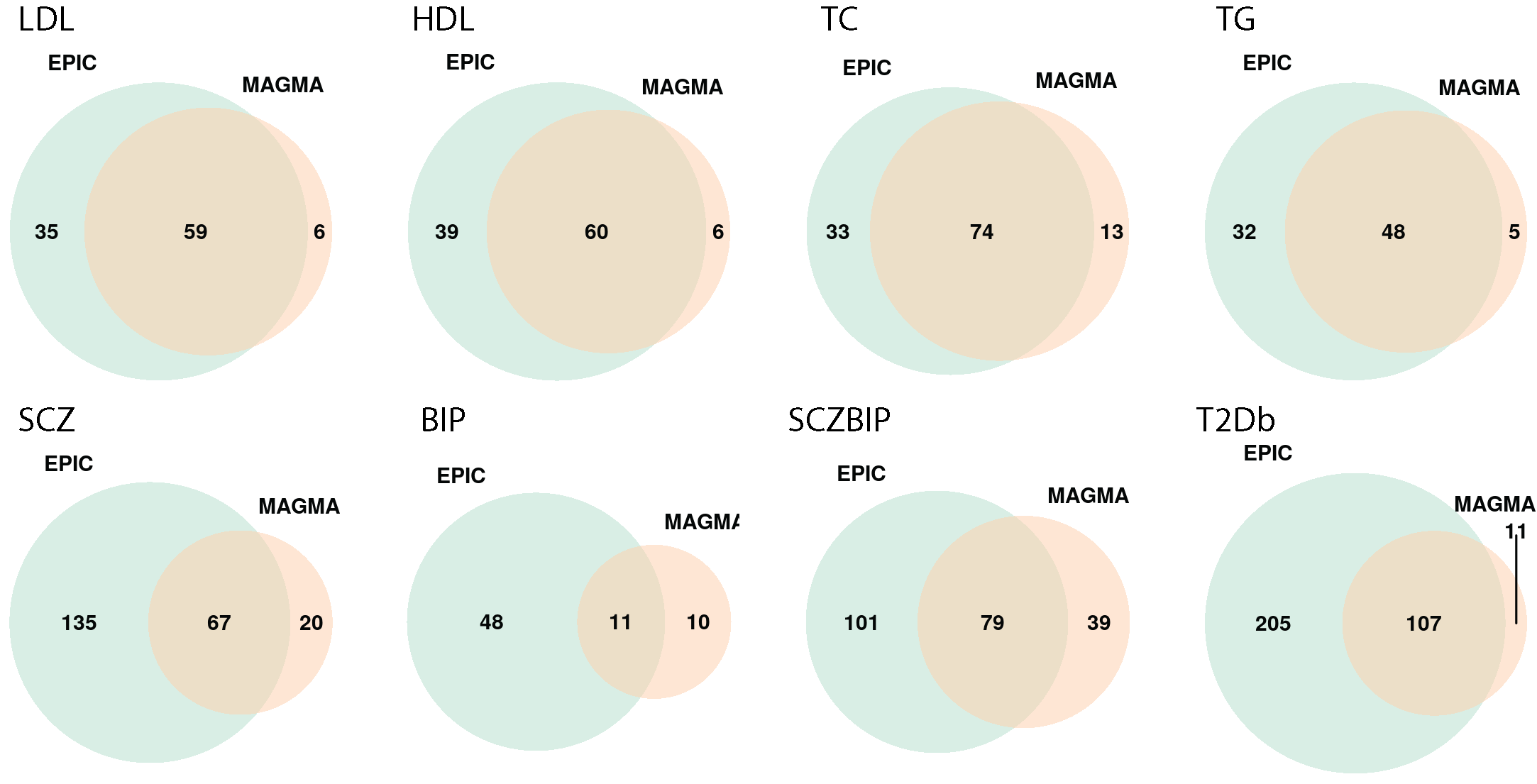


**Fig C: Gene-level association testing results for housekeeping genes.** Human housekeeping genes are obtained from the Housekeeping and Reference Transcript Atlas [11]; the numbers of housekeeping genes included in the boxplot (nominal $p$-values) and the Q-Q (quantile-quantile) plot are included in the title. EPIC obtains better type I error control compared to MAGMA [12, 13] and ACAT [14]. For psychiatric diseases and type 2 diabetes (T2Db), all three methods seemingly show inflated false positive rates, with the top five significantly associated genes shown. Importantly, these genes have been previously reported to be trait relevant: *HSP* gene family [15], *ALDOA* [15], *PPP2R5B* [16], *RMND5A* [17] for SCZ; *RAD23A* [17], *WSB2* [18], *PPP2R5B* [19], *COX5A* [20], and *ADO* [21] for T2Db. This indicates that part of the housekeeping genes, while constitutively expressed to maintain cellular functions, can still be associated with complex traits.

**
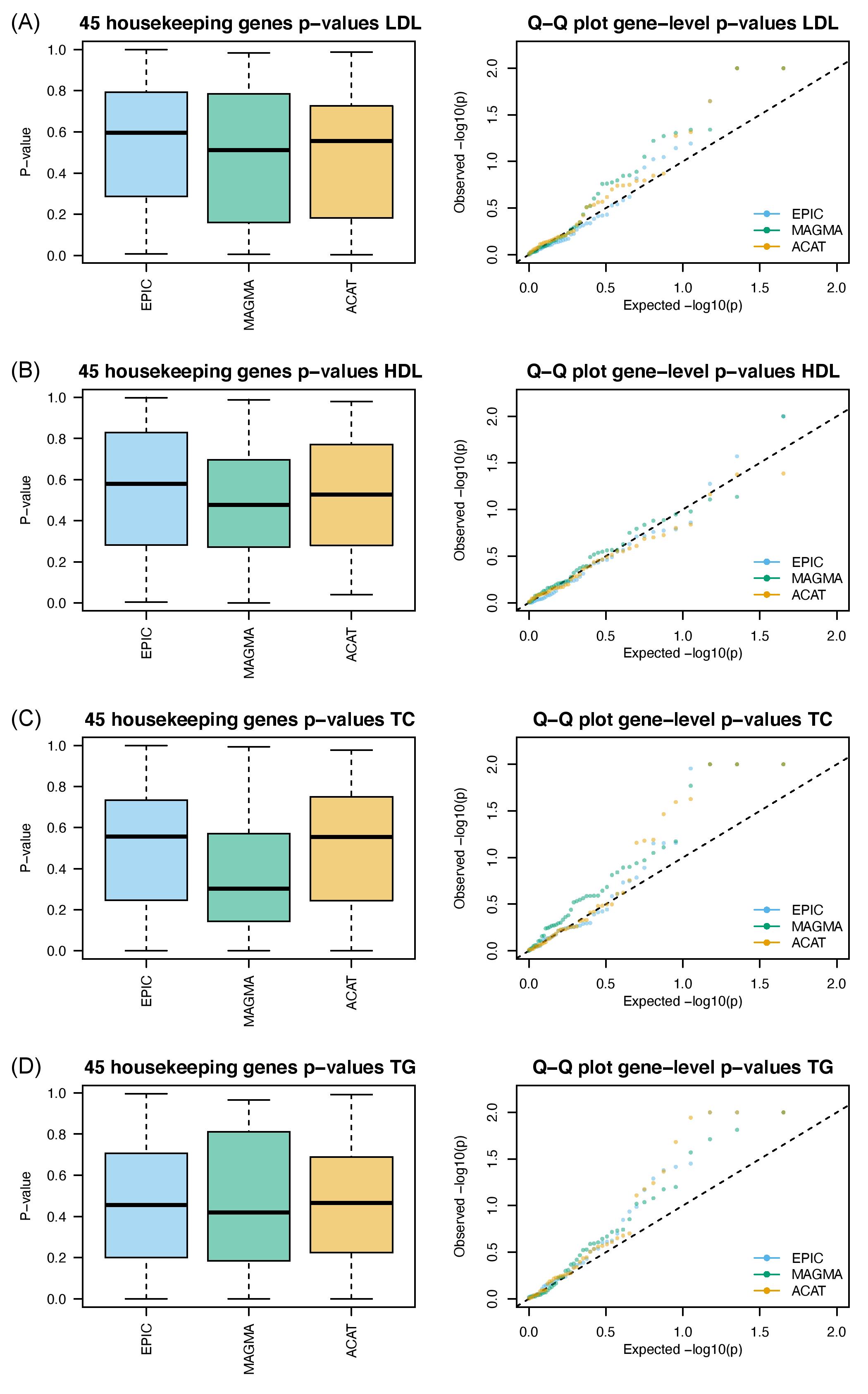
**

**
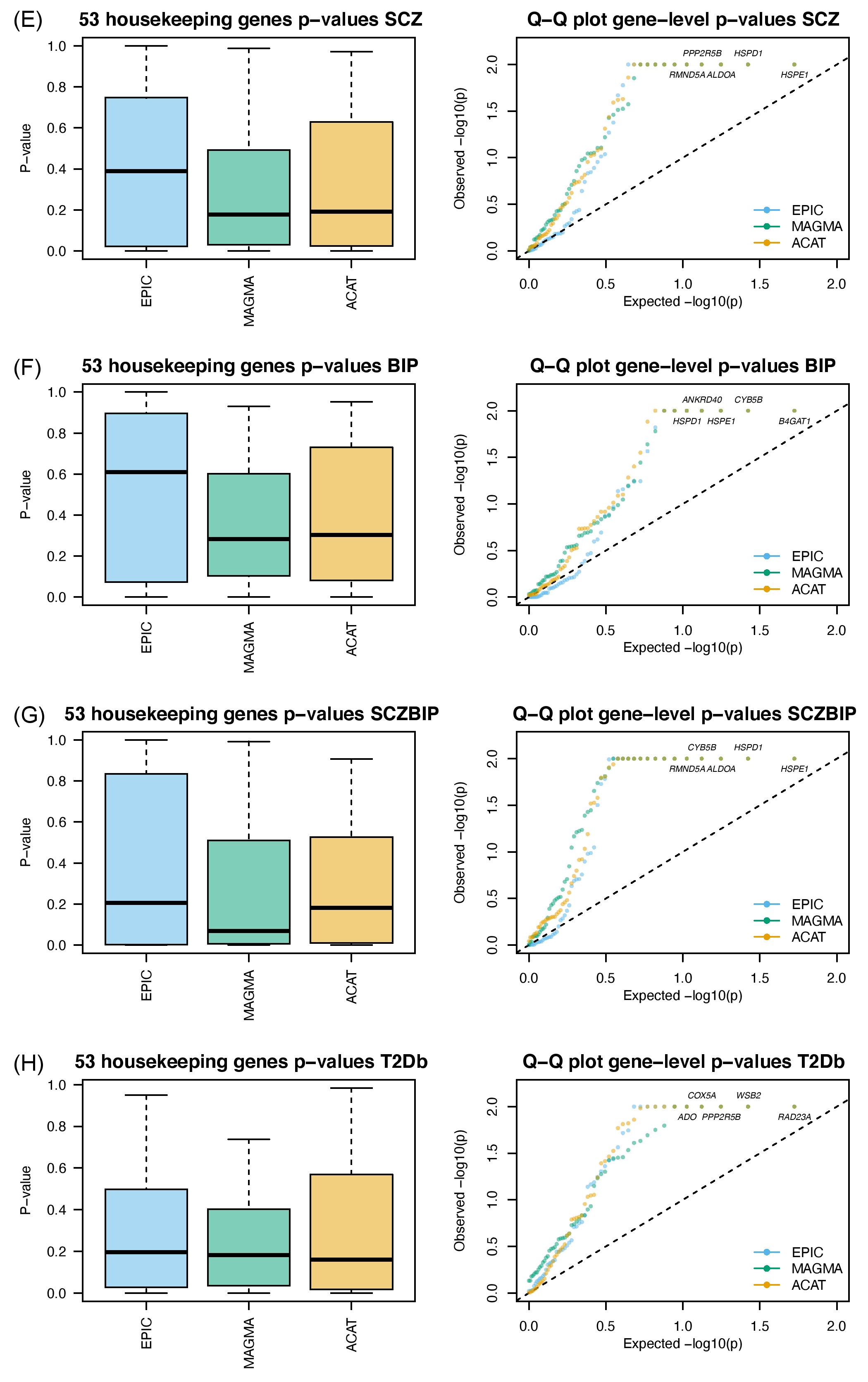
**

**Fig D. UMAP plots of pancreatic islet scRNA-seq datasets.** (A) Baron et al. [8]: UMAP embedding of 8,569 InDrop single-cell profiles from three healthy donors; (B) Segerstolpe et al. [9]: UMAP embedding of 1,068 Smart-seq2 single-cell profiles from six healthy donors.

(A)


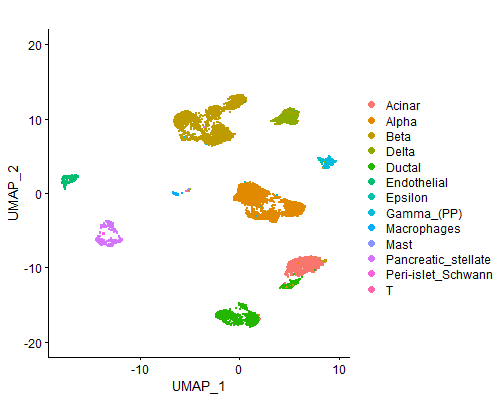


(B)


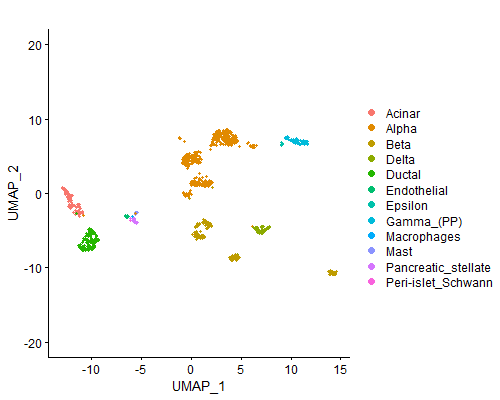


**Fig E. Validation strategies of cell-type enrichment results for schizophrenia.** Volcano plot for 287 differentially expressed (DE) genes that were reported from an independent case versus control study for schizophrenia using bulk RNA-seq.


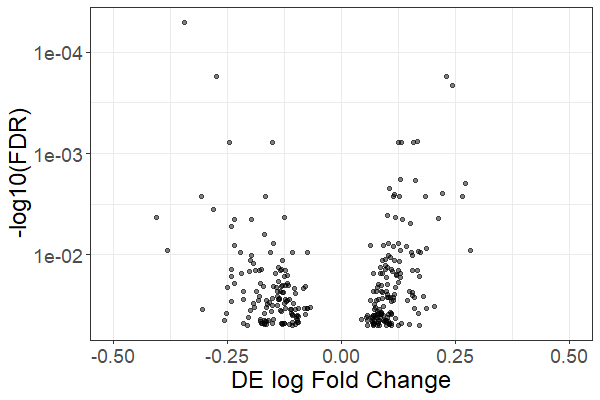


**Fig F: EPIC’s type I error control for gene-level association testing via simulation studies.** We randomly selected 100 genes with well-pruned and annotated common SNPs from the SCZ GWAS, where the number of SNPs per gene ranges from 10 to 195, with mean = 53.2 and median = 31.5. For each gene, we resampled the reference 1000 Genome Project genotype matrix 5000 times from the original 503 individuals. For each individual, we simulated a binary phenotype from a Bernoulli distribution with probabilities 0.1, 0.2, and 0.5. For each SNP, we fit a logistic regression model using the 5,000 samples to generate SNP-level z-scores with corresponding two-sided $p$-values as summary statistics. We repeat the procedure 1,000 times and apply EPIC to carry out gene-level testing. (A) Q-Q plots of $p$-values from the 1,000 runs. (B) EPIC controls for type I error for both small genes (number of SNPs $\leq$ 50) and large genes (number of SNPs $>$ 50). (C) EPIC further achieves a near-zero type I error rate if $p$-values are adjusted for multiple testing by FDR. The same conclusions hold with varying Bernoulli case probabilities.

**
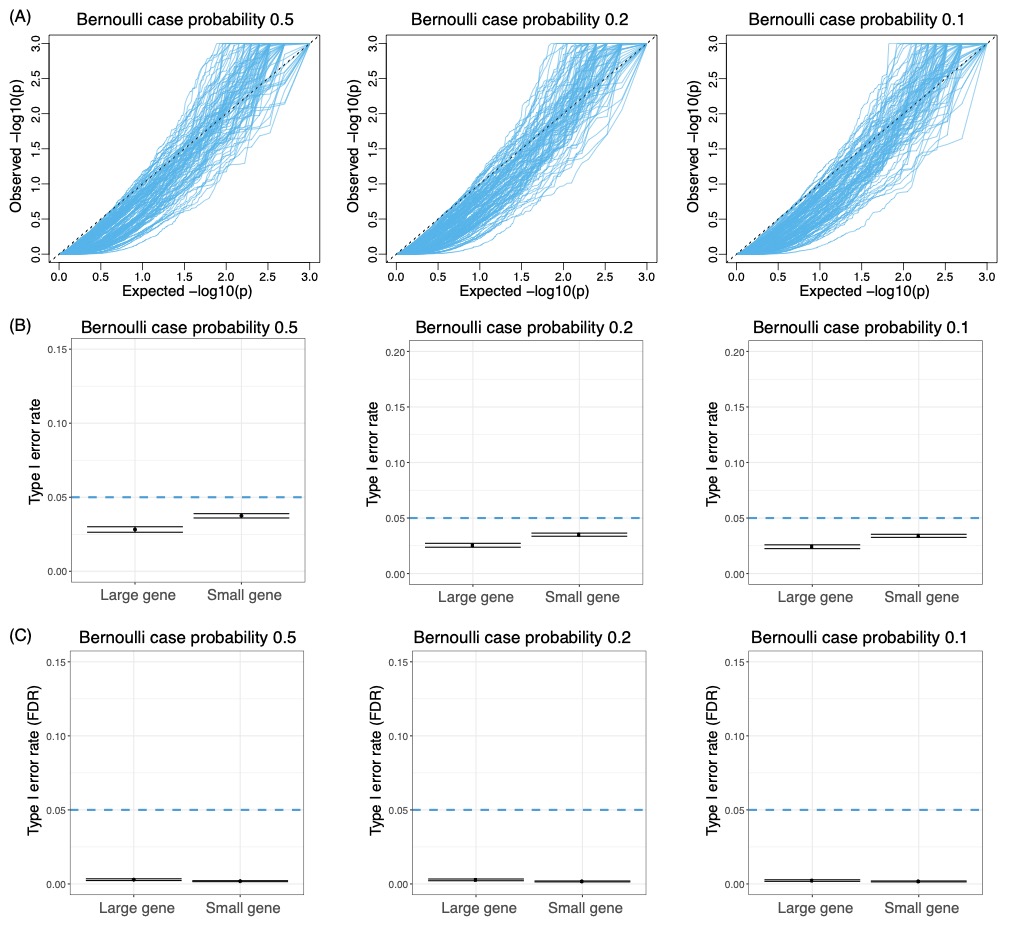
**

**Fig G. EPIC’s type I error control for prioritizing trait-relevant tissues/cell types via permutations.** We randomly permuted the gene expressions to disrupt any correlation structure between gene-level associations and their expression profiles. We applied EPIC to prioritize tissues/cell types: EPIC controls false positives with type I error rates (A) close to zero for the lipid traits, (B) less than 0.02 for the psychiatric diseases, and (C) less than 0.01 for the T2D.

**
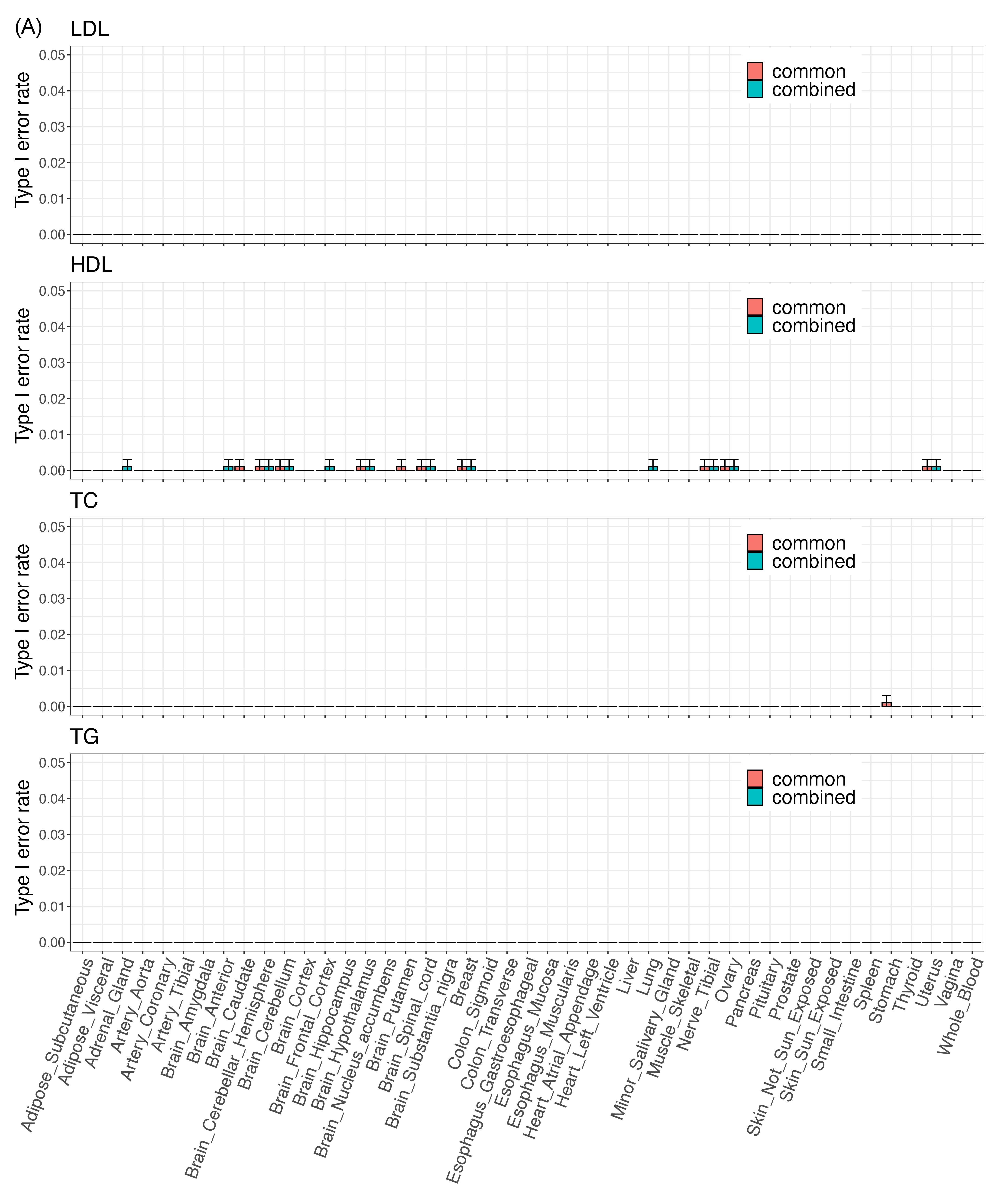
**

**
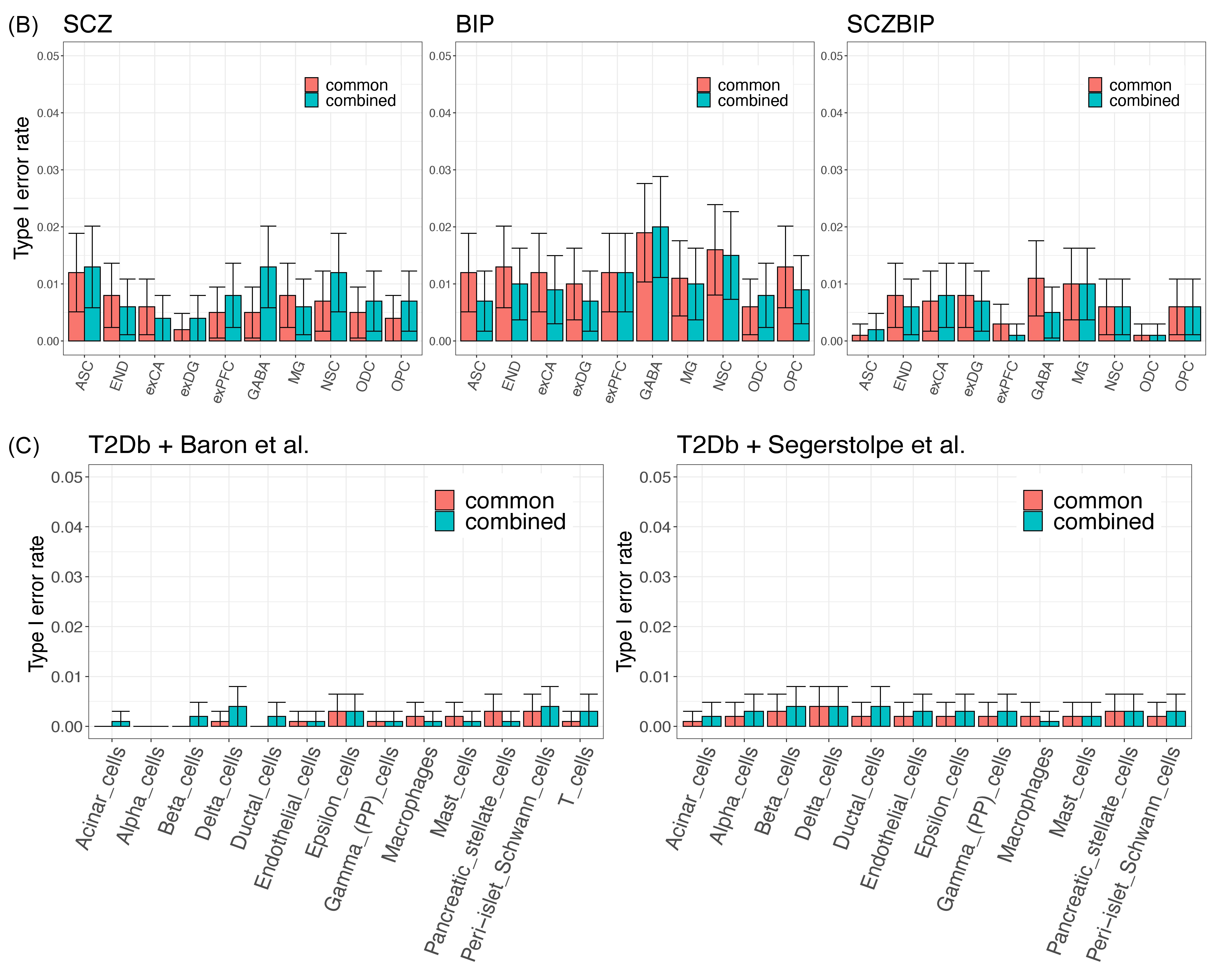
**

**Fig H. Comparison of gene-based *p*-value with different sliding window sizes.** We evaluated the effects of different choices of sliding window size on the gene-level *p*-values. A final set of 8,708 genes are retained in the analysis of the GTEx bulk RNA-seq dataset. There is minimal difference between different window sizes, although computational burden is considerably increased with a larger window. To account for gene-gene correlations and improve computational efficiency, the sliding window size is set to be 10 by EPIC’s default.


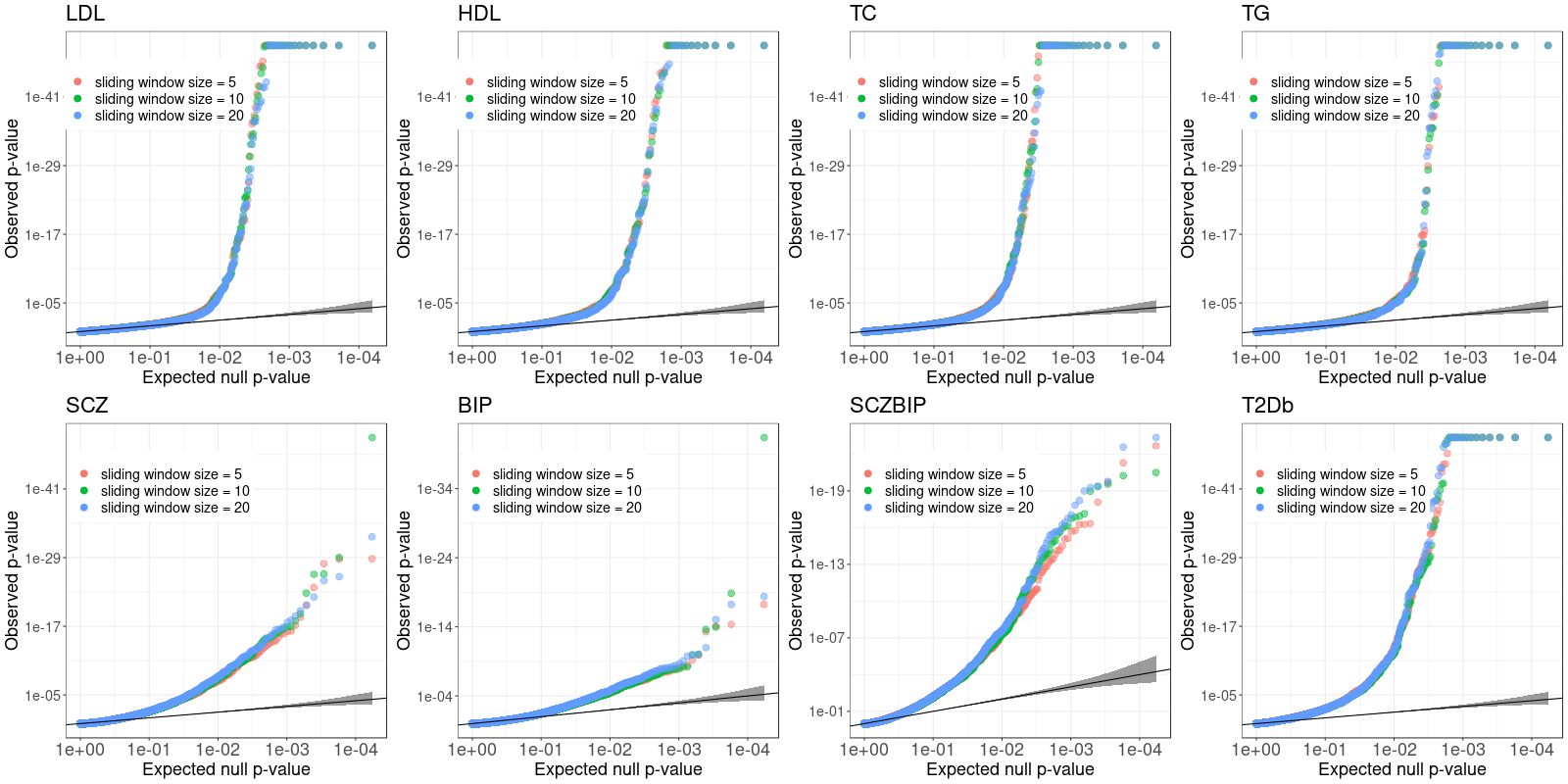


**Fig I. Enrichment results comparison using different MAF cutoffs.** Tissue- or cell-type-specific enrichment results by EPIC (-log(p-value)) with MAF=1% and MAF=0.1% cutoffs. EPIC is robust to the choice of MAF thresholds.


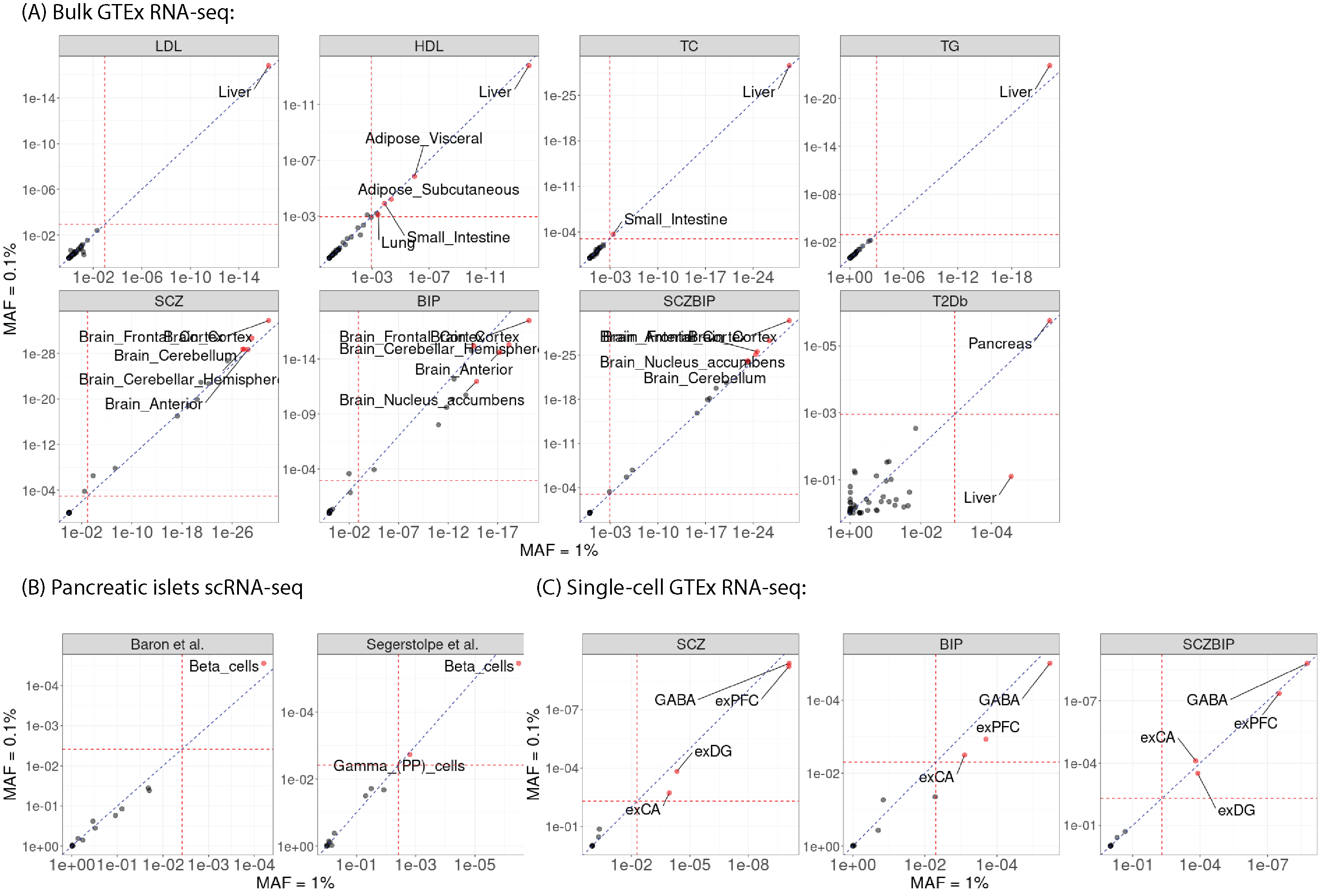


**Fig J. Effect of LD pruning thresholds for common variants.** The left panel shows the percentage of gene-specific LD pruning thresholds on the genome-wide scale. The right panel shows the number of common SNPs per gene across different LD pruning thresholds for genes that need a second-round pruning. The threshold of 0.8 corresponds to the majority of genes with only one-round of pruning. The thresholds less than 0.8 correspond to the remaining genes that need a second round of pruning.

(A) LDL (B) HDL


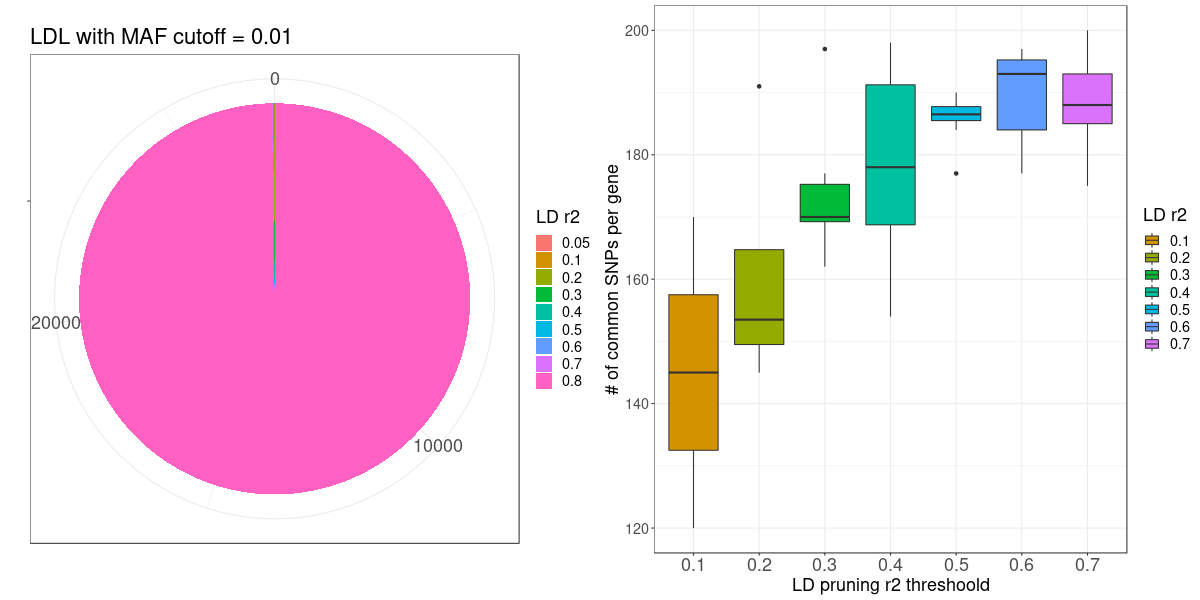

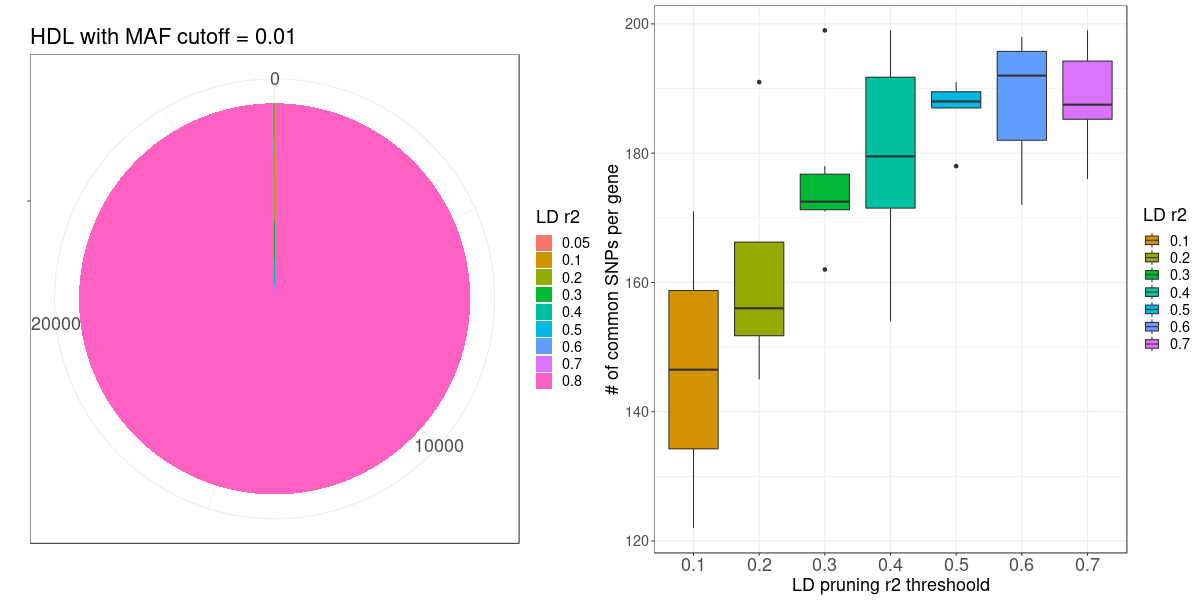


(C) TC (D) TG


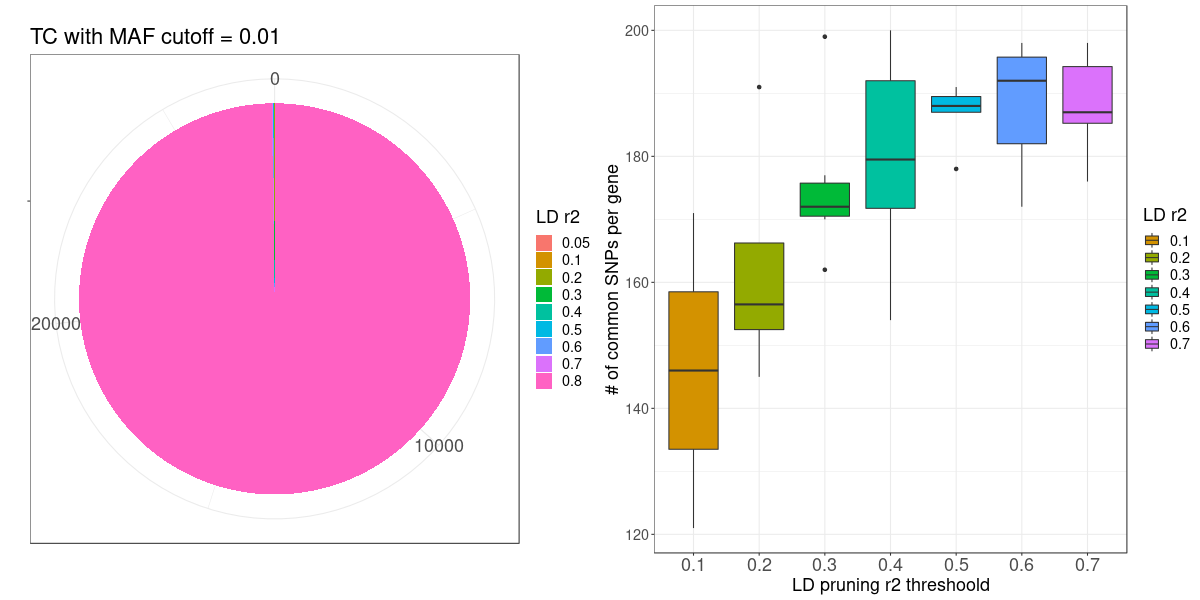

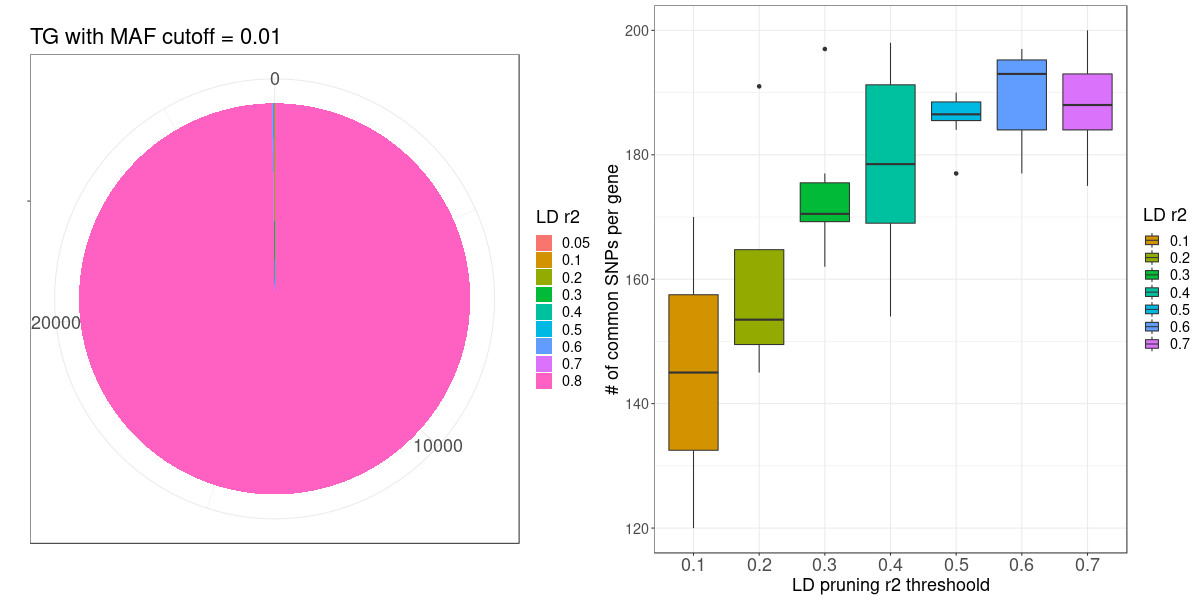


(E) SCZ (F) BIP


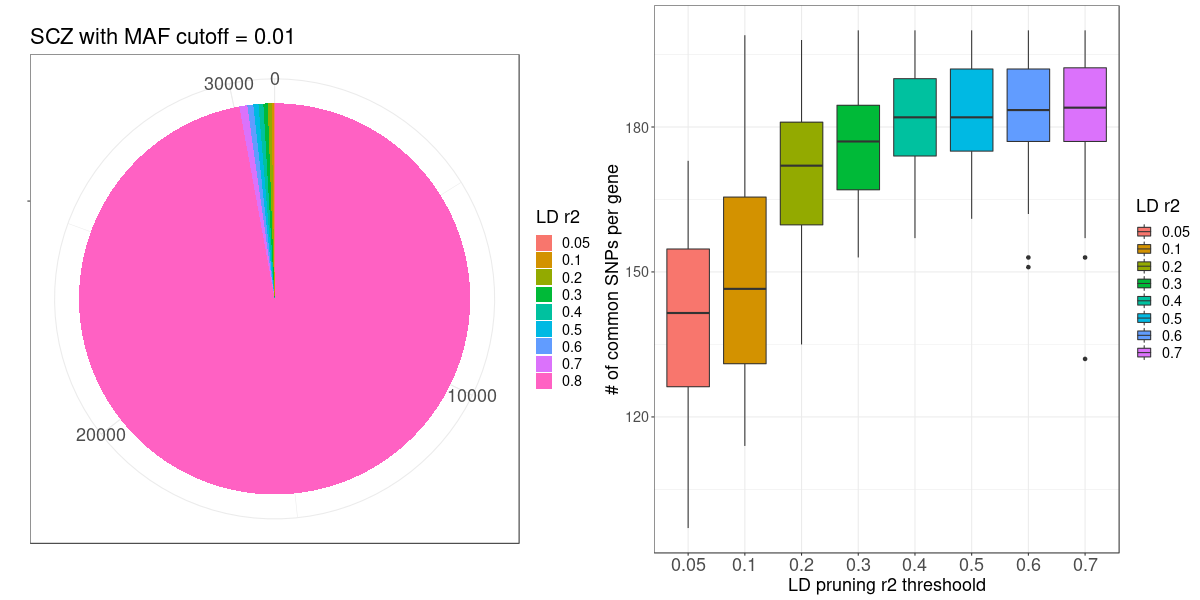

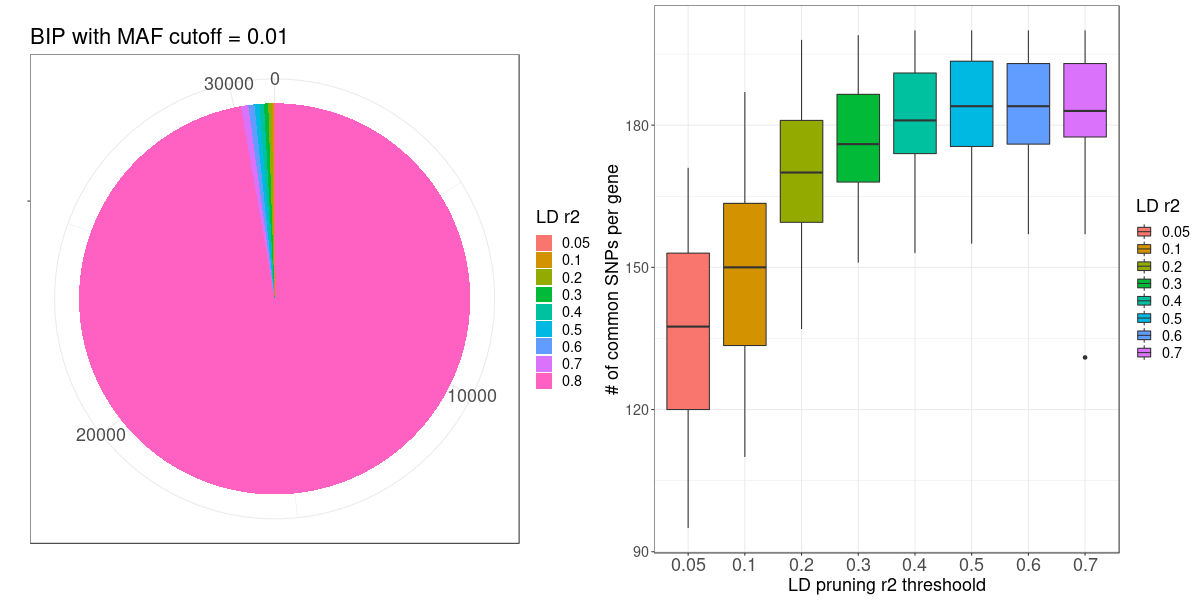


(G) SCZBIP (H) T2Db


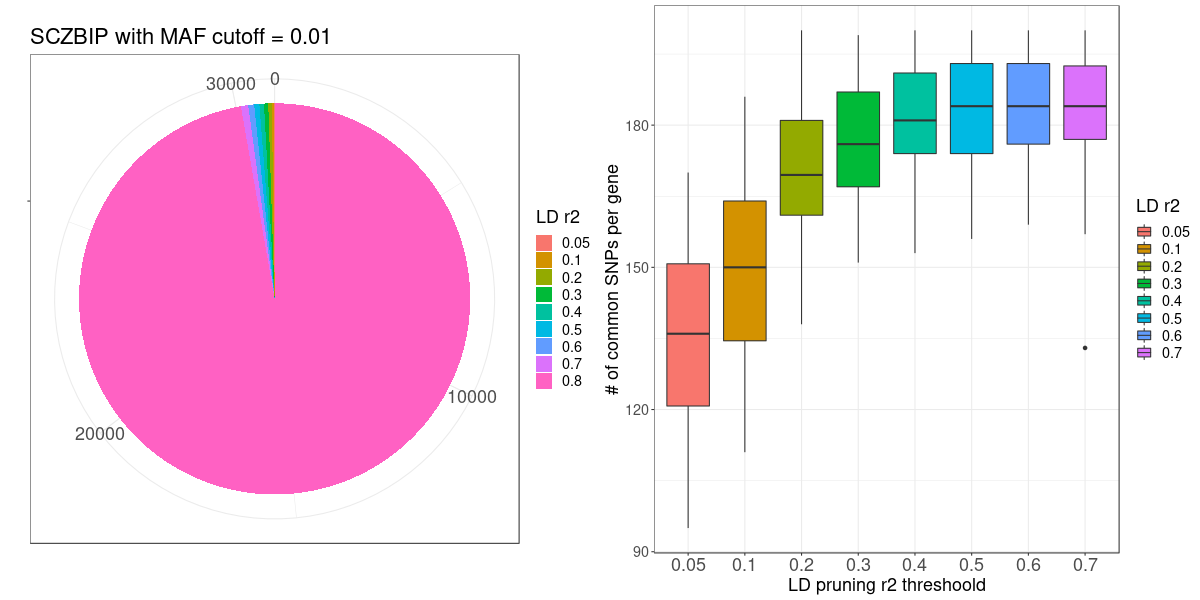

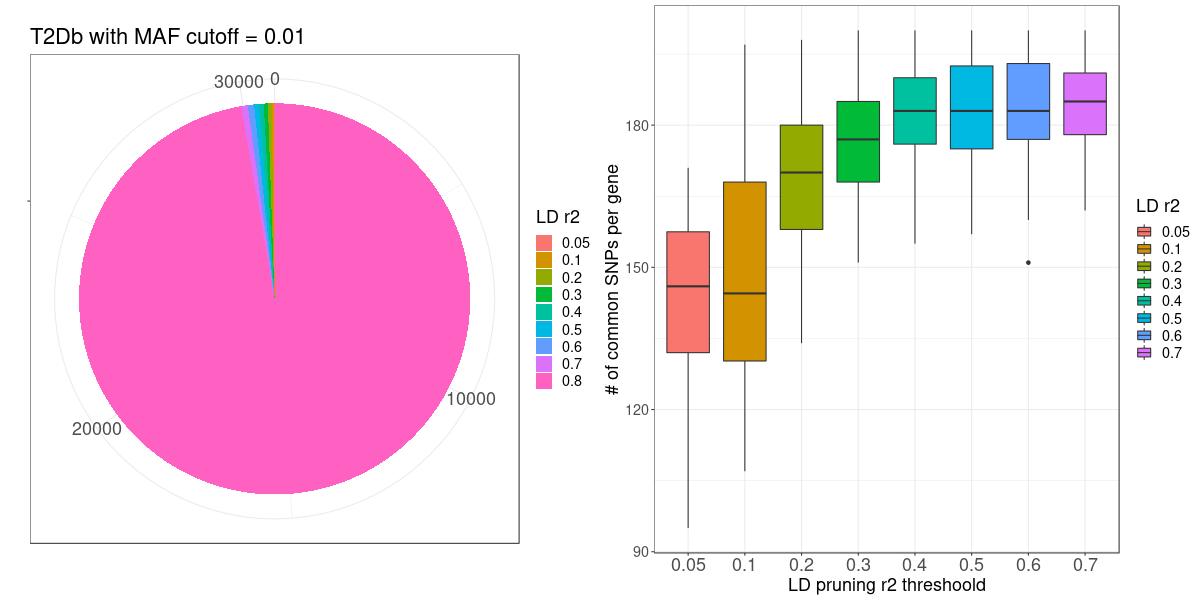


# References

1. Willer CJ, Schmidt EM, Sengupta S, Peloso GM, Gustafsson S, Kanoni S, et al. Discovery and refinement of loci associated with lipid levels. Nat Genet. 2013;45(11):1274-83. Epub 2013/10/08. doi: 10.1038/ng.2797. PubMed PMID: 24097068; PubMed Central PMCID: PMCPMC3838666.

2. Schizophrenia Working Group of the Psychiatric Genomics C. Biological insights from 108 schizophrenia-associated genetic loci. Nature. 2014;511(7510):421-7. Epub 2014/07/25. doi: 10.1038/nature13595. PubMed PMID: 25056061; PubMed Central PMCID: PMCPMC4112379.

3. Pardinas AF, Holmans P, Pocklington AJ, Escott-Price V, Ripke S, Carrera N, et al. Common schizophrenia alleles are enriched in mutation-intolerant genes and in regions under strong background selection. Nat Genet. 2018;50(3):381-9. Epub 2018/02/28. doi: 10.1038/s41588-018-0059-2. PubMed PMID: 29483656; PubMed Central PMCID: PMCPMC5918692.

4. Stahl EA, Breen G, Forstner AJ, McQuillin A, Ripke S, Trubetskoy V, et al. Genome-wide association study identifies 30 loci associated with bipolar disorder. Nat Genet. 2019;51(5):793-803. Epub 2019/05/03. doi: 10.1038/s41588-019-0397-8. PubMed PMID: 31043756; PubMed Central PMCID: PMCPMC6956732.

5. Bipolar D, Schizophrenia Working Group of the Psychiatric Genomics Consortium. Electronic address drve, Bipolar D, Schizophrenia Working Group of the Psychiatric Genomics C. Genomic Dissection of Bipolar Disorder and Schizophrenia, Including 28 Subphenotypes. Cell. 2018;173(7):1705-15 e16. Epub 2018/06/16. doi: 10.1016/j.cell.2018.05.046. PubMed PMID: 29906448; PubMed Central PMCID: PMCPMC6432650.

6. Mahajan A, Taliun D, Thurner M, Robertson NR, Torres JM, Rayner NW, et al. Fine-mapping type 2 diabetes loci to single-variant resolution using high-density imputation and islet-specific epigenome maps. Nat Genet. 2018;50(11):1505-13. Epub 2018/10/10. doi: 10.1038/s41588-018-0241-6. PubMed PMID: 30297969; PubMed Central PMCID: PMCPMC6287706.

7. Consortium GT. The GTEx Consortium atlas of genetic regulatory effects across human tissues. Science. 2020;369(6509):1318-30. Epub 2020/09/12. doi: 10.1126/science.aaz1776. PubMed PMID: 32913098; PubMed Central PMCID: PMCPMC7737656.

8. Baron M, Veres A, Wolock SL, Faust AL, Gaujoux R, Vetere A, et al. A Single-Cell Transcriptomic Map of the Human and Mouse Pancreas Reveals Inter- and Intra-cell Population Structure. Cell Syst. 2016;3(4):346-60 e4. Epub 2016/10/28. doi: 10.1016/j.cels.2016.08.011. PubMed PMID: 27667365; PubMed Central PMCID: PMCPMC5228327.

9. Segerstolpe A, Palasantza A, Eliasson P, Andersson EM, Andreasson AC, Sun X, et al. Single-Cell Transcriptome Profiling of Human Pancreatic Islets in Health and Type 2 Diabetes. Cell Metab. 2016;24(4):593-607. Epub 2016/09/27. doi: 10.1016/j.cmet.2016.08.020. PubMed PMID: 27667667; PubMed Central PMCID: PMCPMC5069352.

10. Habib N, Avraham-Davidi I, Basu A, Burks T, Shekhar K, Hofree M, et al. Massively parallel single-nucleus RNA-seq with DroNc-seq. Nat Methods. 2017;14(10):955-8. Epub 2017/08/29. doi: 10.1038/nmeth.4407. PubMed PMID: 28846088; PubMed Central PMCID: PMCPMC5623139.

11. Hounkpe BW, Chenou F, de Lima F, De Paula EV. HRT Atlas v1.0 database: redefining human and mouse housekeeping genes and candidate reference transcripts by mining massive RNA-seq datasets. Nucleic Acids Res. 2021;49(D1):D947-D55. Epub 2020/07/15. doi: 10.1093/nar/gkaa609. PubMed PMID: 32663312; PubMed Central PMCID: PMCPMC7778946.

12. de Leeuw CA, Mooij JM, Heskes T, Posthuma D. MAGMA: generalized gene-set analysis of GWAS data. PLoS Comput Biol. 2015;11(4):e1004219. Epub 2015/04/18. doi: 10.1371/journal.pcbi.1004219. PubMed PMID: 25885710; PubMed Central PMCID: PMCPMC4401657.

13. Watanabe K, Umicevic Mirkov M, de Leeuw CA, van den Heuvel MP, Posthuma D. Genetic mapping of cell type specificity for complex traits. Nat Commun. 2019;10(1):3222. Epub 2019/07/22. doi: 10.1038/s41467-019-11181-1. PubMed PMID: 31324783; PubMed Central PMCID: PMCPMC6642112.

14. Liu Y, Chen S, Li Z, Morrison AC, Boerwinkle E, Lin X. ACAT: A Fast and Powerful p Value Combination Method for Rare-Variant Analysis in Sequencing Studies. Am J Hum Genet. 2019;104(3):410-21. Epub 2019/03/09. doi: 10.1016/j.ajhg.2019.01.002. PubMed PMID: 30849328; PubMed Central PMCID: PMCPMC6407498.

15. Guan J, Cai JJ, Ji G, Sham PC. Commonality in dysregulated expression of gene sets in cortical brains of individuals with autism, schizophrenia, and bipolar disorder. Transl Psychiatry. 2019;9(1):152. Epub 2019/05/28. doi: 10.1038/s41398-019-0488-4. PubMed PMID: 31127088; PubMed Central PMCID: PMCPMC6534650.

16. Devor A, Andreassen OA, Wang Y, Maki-Marttunen T, Smeland OB, Fan CC, et al. Genetic evidence for role of integration of fast and slow neurotransmission in schizophrenia. Mol Psychiatry. 2017;22(6):792-801. Epub 2017/03/30. doi: 10.1038/mp.2017.33. PubMed PMID: 28348379; PubMed Central PMCID: PMCPMC5495879.

17. Das S, Haq S, Ramakrishna S. Scaffolding protein RanBPM and its interactions in diverse signaling pathways in health and disease. Discov Med. 2018;25(138):177-94. Epub 2018/05/04. PubMed PMID: 29723489.

18. Bugliani M, Liechti R, Cheon H, Suleiman M, Marselli L, Kirkpatrick C, et al. Microarray analysis of isolated human islet transcriptome in type 2 diabetes and the role of the ubiquitin–proteasome system in pancreatic beta cell dysfunction. Molecular and cellular endocrinology. 2013;367(1-2):1-10.

19. Beg M, Srivastava A, Shankar K, Varshney S, Rajan S, Gupta A, et al. PPP2R5B, a regulatory subunit of PP2A, contributes to adipocyte insulin resistance. Mol Cell Endocrinol. 2016;437:97-107. Epub 2016/08/16. doi: 10.1016/j.mce.2016.08.016. PubMed PMID: 27521959.

20. Lu H, Yang Y, Allister EM, Wijesekara N, Wheeler MB. The identification of potential factors associated with the development of type 2 diabetes: a quantitative proteomics approach. Mol Cell Proteomics. 2008;7(8):1434-51. Epub 2008/05/02. doi: 10.1074/mcp.M700478-MCP200. PubMed PMID: 18448419; PubMed Central PMCID: PMCPMC2500228.

21. Ueki I, Stipanuk MH. 3T3-L1 adipocytes and rat adipose tissue have a high capacity for taurine synthesis by the cysteine dioxygenase/cysteinesulfinate decarboxylase and cysteamine dioxygenase pathways. J Nutr. 2009;139(2):207-14. Epub 2008/12/25. doi: 10.3945/jn.108.099085. PubMed PMID: 19106324; PubMed Central PMCID: PMCPMC2635524.
