# Supplementary material for: EPIC: Inferring relevant cell types for complex traits by integrating genome-wide association studies and single-cell RNA sequencing
Source: PLoS Genet. 2022 Jun 16;18(6):e1010251. doi: 10.1371/journal.pgen.1010251 (PMC9242467; doi:10.1371/journal.pgen.1010251)
Supplement: Attachment — Submitted filename: reviews.pdf [file pgen.1010251.s002.pdf]

Wang et al have proposed a statistical framework to identify the tissue-type or cell-type relevant to a complex trait. The method correlates gene-level association statistics with tissue/cell-type specific expression to find the tissue/cell-types enriched for GWAS gene-level associations while combining both common and rare variants. The methodology and real data analysis results are interesting. However, the paper lacks adequate simulation study without which it is challenging to evaluate the merits of the method in comparison with the exiting methods. Below are my specific comments.

1. The study on controlling the type 1 error rate needs to be expanded. Currently it is very limited. How is the type 1 error rate controlled while identifying the tissue/cell type when the gene-level association testing is performed combining both common and rare variants? It is also not clear whether the strategy of combining common and rare variants in gene-level association testing controls the type 1 error rate when testing for gene-level associations. So, a simulation study on this perspective is also needed.
2. I wonder why the authors skipped a formal power comparison study in simulations. Since Magma is the primary method of comparison, an appropriate power comparison between EPIC and MAGMA based on simulations will provide better insight of the method. Even though real data results suggest higher power of EPIC, we do not know the truth in real data.
3. Line 436: In the main model, baseline expression has an effect. What happens if we consider (gamma_c – gamma_A) instead of gamma_c. Intuitively, it makes sense to consider the excess or lack of cell-type specific expression compared to the baseline expression while testing for enrichment.
4. Line 327: the Z-score covariance matrix is simply approximated by LD matrix. But there are other more formal approaches for doing this in the literature. Did the authors explore these?
5. More justification is needed to explain the choices of approximations used in deriving the burden test statistics for a gene based on the summary statistics for the rare variants.
6. The influence test should be elaborated more with references. Selection accuracy of the set of tissue-specific genes important for the complex trait should be evaluated in simulations also.
7. Pubmed correlations are suggestive, but I would not claim it as strong in many cases.
8. Introduction: it is not clear how the methods RolyPoly and LDSC-SEG lack biological interpretations.
9. It is difficult to conclude anything from Fig 5E. The overlap can simply be due to chance.
10. It seems that combining common+rare variants in EPIC leads to better result in some of the validation examples based on real data. This can be clearly pointed out in the results section.
