# Supplementary material for: EPIC: Inferring relevant cell types for complex traits by integrating genome-wide association studies and single-cell RNA sequencing
Source: PLoS Genet. 2022 Jun 16;18(6):e1010251. doi: 10.1371/journal.pgen.1010251 (PMC9242467; doi:10.1371/journal.pgen.1010251)
Supplement: Attachment — Submitted filename: Reviewer_Comments_r2.1.pdf [file pgen.1010251.s003.pdf]

Below are our point-by-point responses to the reviewers' comments. The original reviewers' comments are in black, and our responses are in blue. The changes that we made are highlighted in the main manuscript and supplements.

Reviewer #1:

Genome-wide association studies (GWASs) have yielded genetic variants associated with various complex traits. Emerging evidence suggests that the function of trait associated variants likely acts in a tissue- or cell-type-specific fashion. For many complex traits, however, the specific cell or tissue types leading to risk are unknown. Recent advances of single-cell RNA sequencing (scRNA-seq) provide unprecedented opportunities, alongside challenges, to systematically investigate the cell-type-specific enrichment of GWAS risk variants. Authors propose EPIC, a statistical framework that relates large-scale GWAS summary statistics to cell-type-specific transcriptomic measurements from scRNA-seq data to prioritize trait-relevant cell types. They use known trait-relevant tissues and cell types as ground truths for benchmark, adopt independent GWAS and scRNA-seq datasets for reproducibility, and refer to PubMed keyword search and existing case-control studies for validation. Such an integrative analysis helps elucidate the underlying cell-type-specific disease etiology and prioritize important risk variants. I have following comments.

1. The idea of this manuscript is not novel. It is not the first that relates GWAS results with scRNA-seq datasets. In Watanabe et al. [1], they have already proposed almost the same framework to connect GWAS signals with cell-type-specific from scRNA-seq datasets, where they first converted SNP-based summary statistics into gene-based ones using MAGMA and using the processed single-cell cell-type-specific transcriptome datasets to associate with gene-based Z-scores in a fashion similar to the one on line 436. It is not clear what the major difference between these two methods. Authors should clearly state and discuss it.

> We have rewritten the Introduction section to clarify and highlight the differences between EPIC and MAGMA. The main advantages lie in four folds. First, MAGMA combines the SNP-level p-values to obtain a gene-level p-value using the Brown's approximation – the two-sided tests result in excessive false positives, and the method replaces the correlation coefficient with its square, which acts as a rough correction. Second, EPIC, to our best knowledge, is the first method to separately and jointly model common and rare variants when integrating GWAS and single-cell sequencing data. Third, MAGMA approximates the gene-gene correlations as the correlations between the model sum of squares from the second-step gene-property analysis. However, the gene-gene correlation of the effect sizes should be a function of the LD scores (i.e., the correlations between the SNPs within the genes). EPIC properly accounts for the gene-gene correlation with scalable computation. Last but not least, we propose a cell-type- and gene-specific influential testing scheme to identify genes and gene sets that are relevant to the enrichment, allowing for downstream analysis that goes beyond the tissue and cell type level.

2. In my view, the contribution of this manuscript is that they derive a gene-based test for common and rare variants. I have few questions regarding this. First, how LD can be estimated using reference panel for rare variants? The pseudo-SNP strategy depends on the Burden test, where it assumes that all variants have the same direction of effects. The authors need to show the effectiveness of using this as surrogate.

> In order to provide a good estimation of LD for rare variants, the number of subjects on the reference panel should be large. Hu et al. (*American Journal of Human Genetics*, 2013) rigorously justified the use of an external reference to estimate the correlation matrix of the univariate (i.e., single-variant) test statistics. It showed that, although the LD for the very rare variants might not be estimated well from the external panel, the burden test based on the LD estimates from the external panel performed well. We inherited the same framework to construct the gene-level burden statistics and cited this paper in the "Gene-level association for rare variants" section.
>
> The burden test is one of the most commonly adopted strategies for detecting rare variant associations. The reviewer is absolutely right in that it will work well if the majority of the variants with large effects are in the same direction. In our case, it's more convenient to use the burden test because it's easier to combine with the common variants. In this revision, we carried out additional benchmark against ACAT (Liu et al., *American Journal of Human Genetics*, 2019), a rare variant association testing method. ACAT directly combines correlated SNP-level p-values via the Cauchy combination test and does not make any assumptions on the direction of the effects. While the rare variant association testing results from ACAT and EPIC's burden test are similar (Supplementary Table S2), EPIC achieved substantially increased statistical power when integrating the common and rare variants through joint modeling of the two chi-square statistics using the pseudo-SNP strategy (Supplementary Table S2; Supplementary Figure S1).

Notably, the type I error control between EPIC and ACAT are on par with each other (Supplementary Figure S3). As pointed out by the third reviewer, "combining common+rare variants in EPIC leads to better result in some of the validation examples based on real data."

EPIC, to our best knowledge, is the first method to separately and jointly model common and rare variants in the integrative analysis of GWAS summary statistics and transcriptomic data. For rare variants, although it is generally underpowered, EPIC successfully identified liver and brain as the top-rank and significant tissue for the lipid traits and the neuropsychiatric disorders (Supplementary Table S4).

Second, genes are not distributed equally across the genome. Some regions are denser and some are sparser. How do we know that a sliding window of size d will capture the major correlated genes?

The sliding windows contain the same number of genes. In Supplementary Figure S8, we evaluated the effects of different sliding window sizes on the gene-level p-values. There is minimal difference between different window sizes, although the computational burden is considerably increased with a larger window. To account for gene-gene correlations and to improve computational efficiency, the sliding window size is set to be 10 by EPIC's default.

3. In the main results, it looks like that at least four out of eight traits (Figure S1) have problem of inflated p-values, especially for SCZ and T2D. Before making any statement of more significant findings, one need to demonstrate its type I error control. Authors only use housekeeping genes to show this point but I cannot find the nominal p-value or qq-plot. It is insufficient to claim that the method achieves well-controlled type I error.

To evaluate EPIC's type I error control, we carried out, in this revision, the following tasks.

(i) In the Q-Q plots shown in Supplementary Figure S1, we took the negative log transformation of the observed and expected p-values, focusing on power assessment. As such, the densities of the p-values are not the same along the x- and y-axis (i.e., most of the null p-values are condensed close to the origin). We generated Q-Q plots for genes with p-values from 0.05 to 1, excluding potential trait-relevant genes. We showed that across all traits, EPIC's p-values are uniformly distributed in this range. Please see updated Supplementary Figure S1 for results.

(ii) In this revision, we have added Q-Q plots for the housekeeping genes in Supplementary Figure S3 and included the number of housekeeping genes in the titles. We show that EPIC obtains better type I error control compared to MAGMA and ACAT. Notably, for psychiatric diseases and type 2 diabetes (T2Db), all three methods seemingly show inflated false-positive rates – we focused on the top five significantly associated genes and confirmed that they had been previously reported to be trait relevant (see Supplementary Figure S3's caption for details).

(iii) We carried out simulation studies to assess EPIC's type I error control for gene-level association testing and tissue and cell-type prioritization. Please see our reply to the third reviewer's first comment.

<u>Reviewer #2:</u>

Wang et al. presented a novel method EPIC that relates large-scale GWAS summary statistics to cell-type-specific gene expression measurements from single-cell RNA sequencing. The novel features of EPIC include a powerful gene-level test statistics for both common and rare variants, and an adaptation of generalized least squares to prioritize trait relevant cell types while accounting for the correlation structures both within and between genes. EPIC is applied to multiple scRNA-seq datasets from different platforms and identify cell types underlying type 2 diabetes and schizophrenia and outperforms existing approaches include LDSC-SEG and RolyPoly. Overall, this is a very well written paper with a nice method. I enjoyed reading the paper and only have a few comments that hopefully will help further improve the quality of the paper:

There are two major advantages of EPIC: (1) it models the correlation structures both within and between genes (while LDSC-SEG and RolyPoly directly model correlation among SNPs), and (2) it uses a new framework to link the gene-level z-scores with gene expression level (while LDSC-SEG and RolyPoly uses evidence for differential gene expression instead of expression level directly). It would be helpful to run simulations to illustrate which advantage explains the majority of EPIC's power gain over LDSC-SEG and RolyPoly. Would it also be possible to disentangle these two advantages in the real data applications?

Thanks for your encouraging comments. We have carried out additional simulation studies to assess EPIC's power and type I error control. Please refer to our responses to the third reviewer's first and second comments.

In this revision, we have also carried out additional benchmark analyses, using the t-statistics from differential gene expression analysis as covariates in the second-step regression. The gene-specific t-statistics is obtained by comparing expression in the cell type of interest v.s. all other cell types, as implemented in the LDSC-SEG method (Finucane et al., *Nature Genetics*, 2018). Our benchmark results suggest that the use of t-statistics in EPIC's framework cannot consistently identify the enriched tissues/cell types as expected. The results across all eight traits are summarized below.

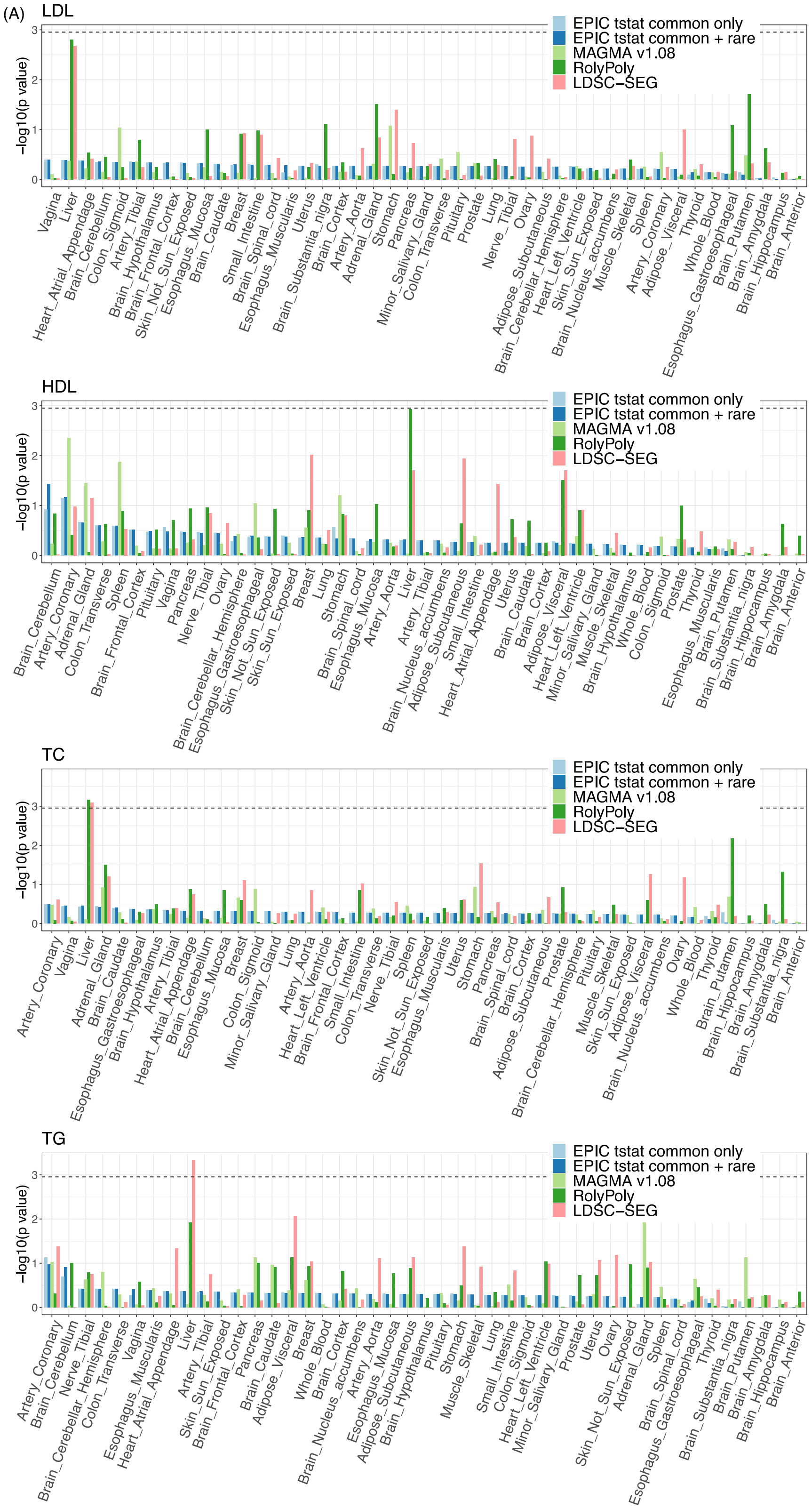

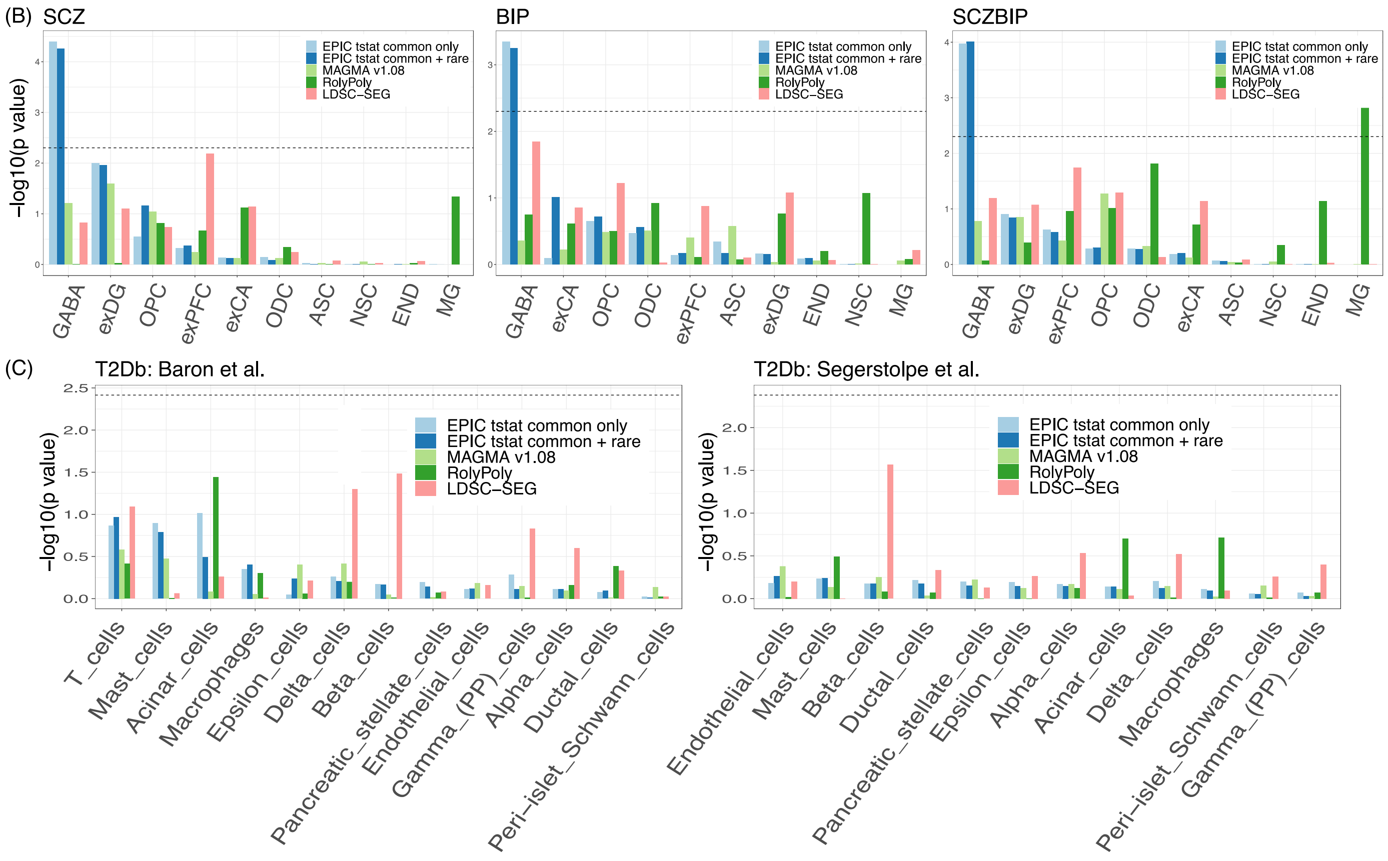

The gene-level association test reminds me of the variance component model used in SKAT. How does the association test in EPIC compare to SKAT, or a similar variance component test that incorporates common variants only, or both common and rare variants?

In this revision, we tried to benchmark against MetaSKAT (Lee et al., *American Journal of Human Genetics*, 2013), a variance component test using summary statistics. However, the R package via CRAN (https://cran.r-project.org/web/packages/MetaSKAT/) has been deprecated. While we were able to install an archived version, we could not generate the MSSD and MInfo input files using only the reference genotype and the summary statistics, without the required original data in PLINK format. We posted our question in the Google group https://groups.google.com/g/skat_slee; unfortunately, we don't think there has been a reply for a year.

As an alternative, we ended up using the Meta-Analysis of Gene-level Associations (MAGA) package (Hu et al., *American Journal of Human Genetics*, 2013), which implemented the SKAT-based test using summary statistics. The Q-Q plots across all traits and results from housekeeping-gene-based evaluations are attached below. We show that while EPIC is more powerful compared to SKAT, the two methods share similar type I error control. Since we were not able to use the default SKAT/MetaSKAT package, we updated Supplementary Figure S1 and Supplementary Figure S3, only showing the benchmark results from EPIC, MAGMA, and a more recent method ACAT (Liu et al., *American Journal of Human Genetics*, 2019).

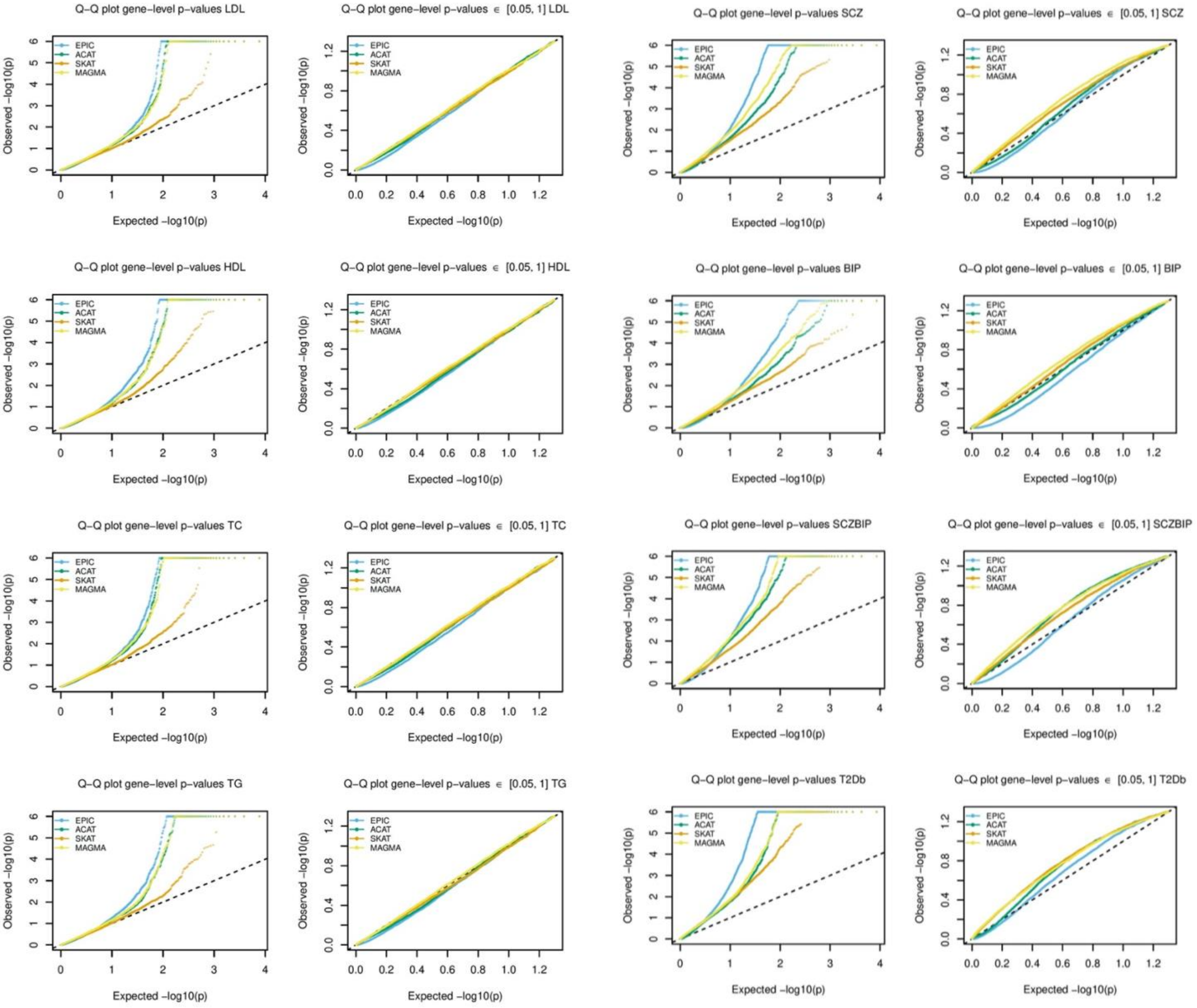

Q−Q plot gene−level p−values LDL
Q−Q plot gene−level p−values ∈ [0.05, 1] LDL
Q−Q plot gene−level p−values SCZ
Q−Q plot gene−level p−values ∈ [0.05, 1] SCZ
Q−Q plot gene−level p−values HDL
Q−Q plot gene−level p−values ∈ [0.05, 1] HDL
Q−Q plot gene−level p−values BIP
Q−Q plot gene−level p−values ∈ [0.05, 1] BIP
Q−Q plot gene−level p−values TC
Q−Q plot gene−level p−values ∈ [0.05, 1] TC
Q−Q plot gene−level p−values SCZBIP
Q−Q plot gene−level p−values ∈ [0.05, 1] SCZBIP
Q−Q plot gene−level p−values TG
Q−Q plot gene−level p−values ∈ [0.05, 1] TG
Q−Q plot gene−level p−values T2Db
Q−Q plot gene−level p−values ∈ [0.05, 1] T2Db
EPIC
ACAT
SKAT
MAGMA
Observed −log10(p)
Expected −log10(p)

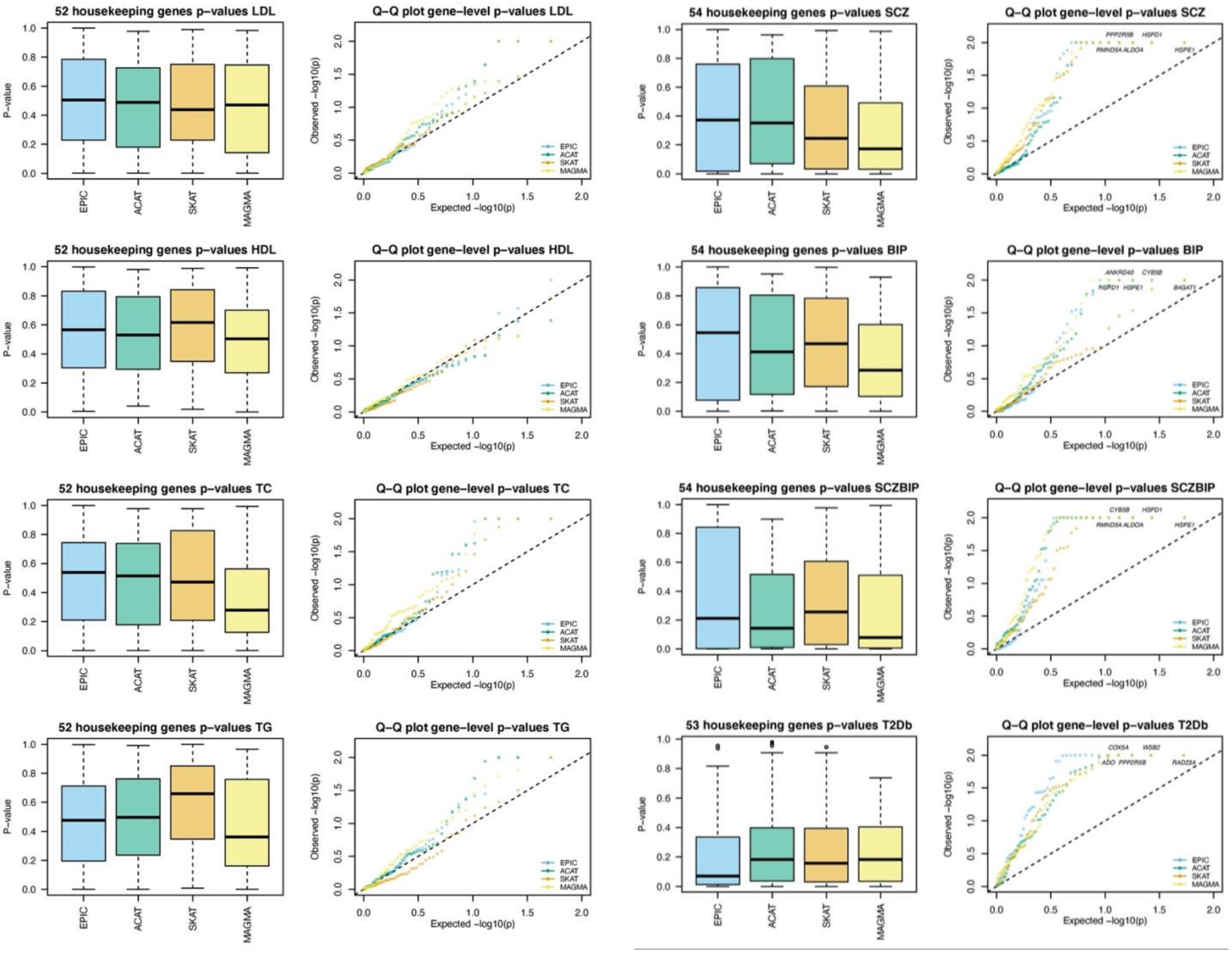

For Figure S3, could you show a qq-plot instead? The figure legend is unclear, and I couldn't figure out how many genes are included in these boxplots (52, 2088, or 8708?). Similarly, for Figure S4, could you show a qq-plot to see whether p-values are calibrated across a range of cutoffs?

Thanks for the suggestions! In this revision, we have added Q-Q plots for the housekeeping genes in Supplementary Figure S3 and included the number of housekeeping genes in the titles. We show that EPIC obtains better type I error control compared to MAGMA and ACAT. Notably, for psychiatric diseases and type 2 diabetes (T2Db), all three methods seemingly show inflated false-positive rates – we focused on the top five significantly associated genes and confirmed that they had been previously reported to be trait relevant (see Supplementary Figure S3's caption for details). This indicates that part of the housekeeping genes, while constitutively expressed to maintain cellular functions, can still be associated with complex traits.

Therefore, for all empirical studies, we generated Q-Q plots for genes with p-values from 0.05 to 1, excluding potential trait-relevant genes. We showed that across all traits, EPIC's p-values are uniformly distributed in this range. See updated Supplementary Figure S1 for results.

We have also updated Supplementary Figure S6 (renumbered in the revision) to include the Q-Q plots for the p-values from simulations. EPIC controls for type I error for both small genes (number of SNPs ≤ 50) and large genes (number of SNPs > 50); EPIC further achieves a near-zero type I error rate if p-values are adjusted for multiple testing by FDR.

Reviewer #3:

Wang et al have proposed a statistical framework to identify the tissue-type or cell-type relevant to a complex trait. The method correlates gene-level association statistics with tissue/cell-type specific expression to find the tissue/cell-types enriched for GWAS gene-level associations while combining both common and rare variants. The methodology and real data analysis results are interesting. However, the paper lacks adequate simulation study without which it is challenging to evaluate the merits of the method in comparison with the exiting methods. Below are my specific comments.

1. The study on controlling the type 1 error rate needs to be expanded. Currently it is very limited. How is the type 1 error rate controlled while identifying the tissue/cell type when the gene-level association testing is performed combining both common and rare variants? It is also not clear whether the strategy of combining common and rare variants in gene-level association testing controls the type 1 error rate when testing for gene-level associations. So, a simulation study on this perspective is also needed.

Thanks! We have added extensive simulation- and permutation-based studies in this revision.

To assess EPIC's type I error control for gene-level association testing, we first carried out simulation studies under the null; in the newly generated Supplementary Figure S6, we show that for both large and small genes (with number of SNPs greater/less than 50) EPIC controls for false positives. We also detailed the simulation studies below in Materials and Methods:

"We first ran a null simulation to evaluate EPIC's type I error control for its gene-level association testing. We generated simulated null genes in the following way. (i) We randomly selected 100 genes with well-pruned and annotated common SNPs from our SCZ GWAS analysis; the number of SNPs per gene ranged from 10 to 195, with a mean of 53.2 and a median of 31.5. (ii) For each gene, we resampled the reference genotype matrix from the 1000 Genomes Project 5,000 times and obtained a resampled reference genotype matrix $X_{n\times m}$, where $n = 5000$ individuals, $m =$ the number of SNPs, and $X_{ij} \in \{0,1,2\}$ indicates the number of effect alleles for individual i and SNP j. (iii) We simulated binary phenotype $Y_i \sim \text{Bernoulli}(0.5)$ for each individual i. For each SNP j, we fit a logistic regression to generate SNP-level z-scores with corresponding two-sided p-values. (iv) To compute the correlation matrix for each gene, we obtained the sparse and shrinkage POET estimators within the sliding window. (v) Repeat (ii)-(iv) 1,000 times and calculate the type I error rate for small genes (number of SNPs ≤ 50) and large genes (number of SNPs > 50)."

To assess EPIC's type I error control for the second-step regression analysis, we randomly permuted the gene expressions to disrupt any correlation structure between gene-level associations and their expression profiles. We applied EPIC to prioritize tissues/cell types: EPIC controls false positives with type I error rates close to zero for the lipid traits, less than 0.02 for the psychiatric diseases, and less than 0.01 for the T2Db (Supplementary Figure S7).

2. I wonder why the authors skipped a formal power comparison study in simulations. Since Magma is the primary method of comparison, an appropriate power comparison between EPIC and MAGMA based on simulations will provide better insight of the method. Even though real data results suggest higher power of EPIC, we do not know the truth in real data.

To assess EPIC's power for gene-level association testing, we generated dichotomous phenotypes as a function of the SNP-level genotypes with varied proportions of causal variants, effect sizes, and directions of effects. We then computed the summary statistics for each SNP, which were used as input for EPIC. The proportion of causal variants was set to be 5%, 20%, and 50%, representative of both sparse and dense signals; effect sizes, as well as directions of effects, were also varied. Altogether, we had a total of 30 simulation configurations for each of the 100 genes. Empirical power was estimated as the proportion of p-values less than $\alpha = 10^{-6}$. The simulation was repeated 1,000 times to allow for standard error estimates. Our results suggest that under 30 different simulation configurations, EPIC is more powerful than MAGMA and that the power gain is substantial when the signals are sparse or in different directions. The simulation details are included in Materials and Methods; the results are summarized in the newly generated Supplementary Table S5.

3. Line 436: In the main model, baseline expression has an effect. What happens if we consider (gamma_c – _gamma_A) instead of gamma_c. Intuitively, it makes sense to consider the excess or lack of cell-type specific expression compared to the baseline expression while testing for enrichment.

We respectfully disagree with the reviewer. The multiple regression framework $Y = \gamma_0 + E_c\gamma_c + A\gamma_A + \epsilon$ tests for the *conditional* association between gene-level GWAS summary statistics Y and cell-type-specific gene expression E_c, fixing baseline gene expression A. In other words, the baseline expression A is controlled for / conditioned upon when testing for a significant γ_c. When E_c and A are separately regressed on Y to test for marginal associations, testing the difference in the corresponding coefficients is the same as testing for γ_c in our current setting.

4. Line 327: the Z-score covariance matrix is simply approximated by LD matrix. But there are other more formal approaches for doing this in the literature. Did the authors explore these?

The covariance/correlation of the standardized z-scores is the same as the correlation of the effect sizes, and both can be estimated using the Pearson correlations between the SNPs (i.e., the LD matrix). This estimation strategy has been adopted by many GWAS methods to date. Hu et al. (*American Journal of Human Genetics*, 2013) rigorously justified the use of an external reference to estimate the correlation matrix of the univariate (i.e., single-variant) test statistics. We inherited the same framework to construct the gene-level burden statistics and cited this paper in the "Gene-level association for rare variants" section.

5. More justification is needed to explain the choices of approximations used in deriving the burden test statistics for a gene based on the summary statistics for the rare variants.

Hu et al. (*American Journal of Human Genetics*, 2013) proposed a framework to perform gene-level tests for rare variants by combining the single-variant summary statistics. The framework can be used to construct burden test for rare variants and is systematically evaluated for both power and type I error control, with a justification on the use of an external reference to estimate the correlation matrix. We inherited the same framework for rare variant testing and cited this paper in the section "Gene-level associations for rare variants" in Materials and Methods.

6. The influence test should be elaborated more with references. Selection accuracy of the set of tissue-specific genes important for the complex trait should be evaluated in simulations also.

We have added more details and inserted references for the influential test – please refer to the section "Prioritizing trait-relevant cell type(s)" in Materials and Methods.

The DFBETA statistics are well established to identify highly influential observations in regression-based frameworks. With all due respect, we don't think a simulation on this technique per se is within the scope of this paper. Additionally, and perhaps more importantly, we don't think that the relationships between GWAS summary statistics and scRNA-seq expression profiles, coupled with high-dimensional covariance structure, can be easily and realistically recapitulated by simulations. Nevertheless, we did carry out validation in empirical studies to confirm the roles of the tissue- and cell-type-specific highly influential genes. Specifically, we carried out KEGG pathways and gene ontology (GO) enrichment analysis and confirmed that for T2Db the beta-cell-specific influential genes are enriched in terms including insulin secretion and glucose metabolism (Figure 3D). We also carried out testing between the highly influential genes and the significant genes that are called by GWAS and scRNA-seq separately. Please see our response to the 9th comment below.

7. Pubmed correlations are suggestive, but I would not claim it as strong in many cases.

For the PubMed validation, we additionally performed a correlation test and included the p-values in the updated Figure 5. We show that the p-values are highly significant for validating the relevant tissues for all traits and the beta cells for T2Db. Although the p-values are insignificant for the cell types prioritized for psychiatric diseases, the correlation coefficients are positive, and, more importantly, the top enriched cell type (i.e., gabaergic interneurons (GABA)), when pairing with the three psychiatric traits, also returned the largest number of PubMed hits. The insignificant p-values are likely due to the limited number of existing single-cell studies on neuropsychiatric diseases via PubMed. Indeed, the cell types that are found to be enriched by EPIC agree with the recent reports (Skene et al., *Nature Genetics*, 2018 and Bryois et al., *Nature Genetics*, 2020), and we additionally

confirmed their roles utilizing existing case-control studies of differential gene expressions related to SCZ risks. Therefore, we believe that, although the p-values are insignificant as shown in Figure 5C, the inferred cell types are enriched for the GWAS traits. Here, we adopted the PubMed search strategy for validation, as proposed by CoCoNet (Shang et al., *PLoS Genetics*, 2019). In the revised manuscript, we have clarified what the PubMed search results (especially in Figure 5C) really tell us.

8. Introduction: it is not clear how the methods RolyPoly and LDSC-SEG lack biological interpretations.

Thanks. RolyPoly captures the effect of the cell-type-specific gene expression on the covariance of GWAS effect sizes, while LDSC-SEG tests for significant contribution of cell-type-specific expression to trait heritability. We find the effect of the cell-type-specific expression on GWAS covariance structure and partitioning heritability not easily interpretable in biology, but we have chosen to tone down in the revision to make it less subjective.

9. It is difficult to conclude anything from Fig 5E. The overlap can simply be due to chance.

We believe that the reviewer referred to Figure 3E, in which we showed the Venn diagram of the significant calls from gene-level GWAS testing, cell-type-specific gene expression analysis, and EPIC's gene- and cell-type-specific influential analysis. In this revision, we carried out a hypergeometric test of significant overlap between each pair of the three call sets. Specifically, let m and n be the number of significant and insignificant genes for testing A, respectively, k be the number of significant genes from testing B, and q be the number of overlapped significant genes between A and B. We aim to test for an enrichment of testing A's significant genes in B – the p-value of enrichment is derived from the hypergeometric distribution using the cumulative distribution function, coded as phyper(q, m, n, k, lower.tail = FALSE) in R.

The p-values are updated in Fig 3E. Highly influential genes from EPIC's integrative analysis framework significantly overlap with genes returned by both gene-level association testing and cell-type-specific gene expression analyses, while the latter two from GWAS and scRNA-seq showed no significant overlap. The influential analysis by EPIC helps prioritize trait-relevant genes in a cell-type-specific manner.

10. It seems that combining common+rare variants in EPIC leads to better result in some of the validation examples based on real data. This can be clearly pointed out in the results section.

Thanks for the suggestion! We have pointed this out in the results section.
