# Supplementary material for: EPIC: Inferring relevant cell types for complex traits by integrating genome-wide association studies and single-cell RNA sequencing
Source: PLoS Genet. 2022 Jun 16;18(6):e1010251. doi: 10.1371/journal.pgen.1010251 (PMC9242467; doi:10.1371/journal.pgen.1010251)

We thank the editors and reviewers for their encouragement and feedback. The original reviewers' comments are in black, and our responses are in blue.

Reviewer #1: The authors answered all my concerns and I don't have further questions.

Thank you!

Reviewer #2: My previous comments were all well addressed.

Thank you!

Reviewer #3: The authors have addressed my comments adequately. One specific comment: while evaluating EPIC's type I error control for its gene-level association testing, the authors chose Bernoulli(0.5) to simulate the case-control phenotype. However, more realistic choices are 0.1, 0.2 in the population. I would suggest expanding the type 1 error rate study for these choices as well.

Thank you! We repeated the null simulations with population case rates 0.1, 0.2, and 0.5 in the Bernoulli distribution. Results are updated in Figure S6 (pasted on the next page): EPIC controls for type I error under all simulation scenarios.

Figure S6: EPIC's type I error control for gene-level association testing via simulation studies. We randomly selected 100 genes with well-pruned and annotated common SNPs from the SCZ GWAS, where the number of SNPs per gene ranges from 10 to 195, with mean = 53.2 and median = 31.5. For each gene, we resampled the reference 1000 Genome Project genotype matrix 5000 times from the original 503 individuals. For each individual, we simulated a binary phenotype from a Bernoulli distribution with probabilities 0.1, 0.2, and 0.5. For each SNP, we fit a logistic regression model using the 5,000 samples to generate SNP-level z-scores with corresponding two-sided p-values as summary statistics. We repeat the procedure 1,000 times and apply EPIC to carry out gene-level testing. (A) Q-Q plots of p-values from the 1,000 runs. (B) EPIC controls for type I error for both small genes (number of SNPs ≤ 50) and large genes (number of SNPs > 50). (C) EPIC further achieves a near-zero type I error rate if p-values are adjusted for multiple testing by FDR. The same conclusions hold with varying Bernoulli case probabilities.

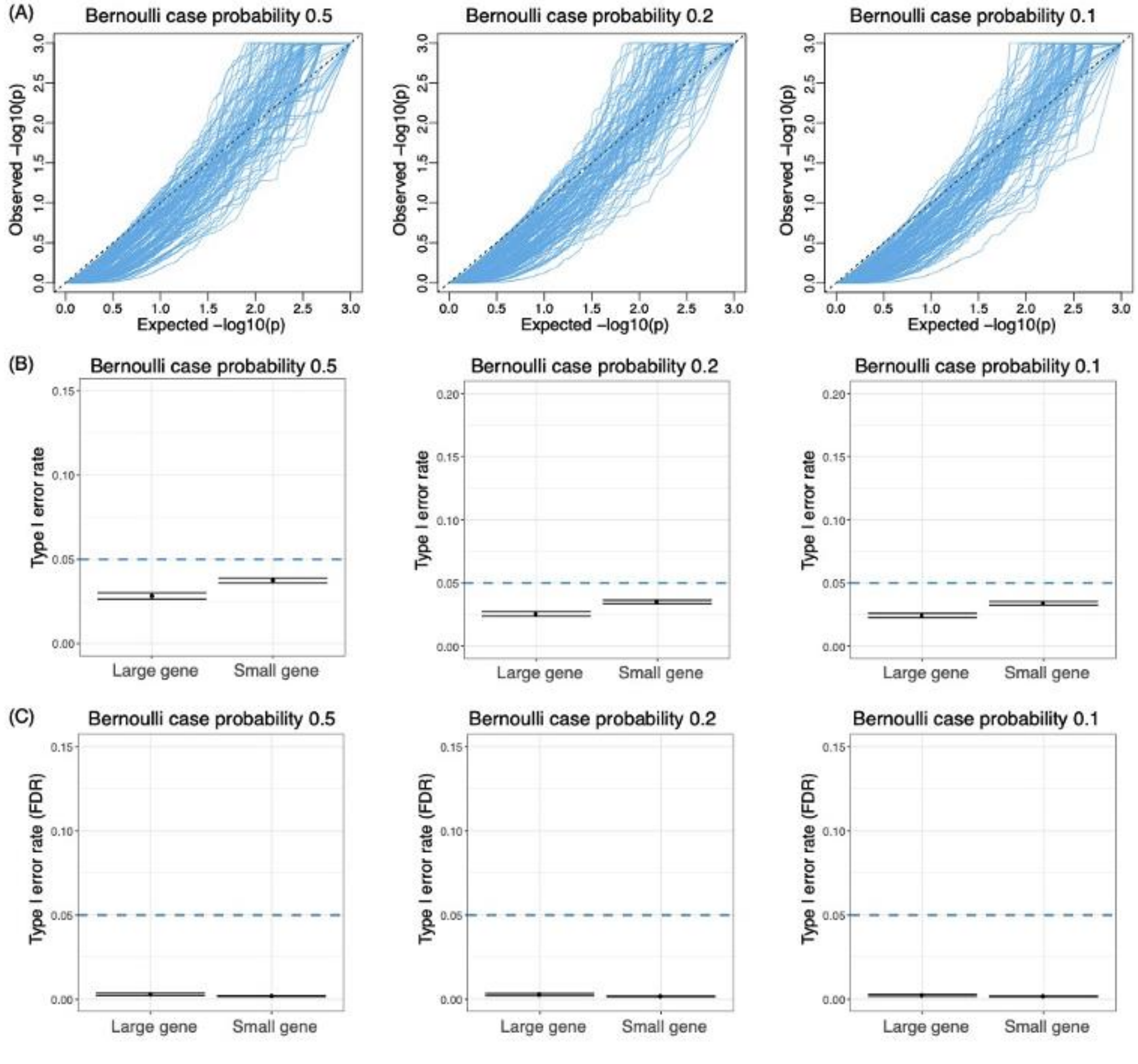

Supplement: Attachment — Submitted filename: Reviewer_Comments_round2.pdf [file pgen.1010251.s004.pdf]
